# Identification of O-GlcNAc Modification Targets in Mouse Retinal Pericytes: Implication of p53 in Pathogenesis of Diabetic Retinopathy

**DOI:** 10.1371/journal.pone.0095561

**Published:** 2014-05-01

**Authors:** Zafer Gurel, Balyn W. Zaro, Matthew R. Pratt, Nader Sheibani

**Affiliations:** 1 Department of Ophthalmology and Visual Sciences, University of Wisconsin, School of Medicine and Public Health, Madison, Wisconsin, United States of America; 2 McPherson Eye Research Institute, University of Wisconsin, School of Medicine and Public Health, Madison, Wisconsin, United States of America; 3 Departments of Chemistry and Molecular and Computational Biology, University of Southern California, Los Angeles, California, United States of America; Bristol Heart Institute, University of Bristol, United Kingdom

## Abstract

Hyperglycemia is the primary cause of the majority of diabetes complications, including diabetic retinopathy (DR). Hyperglycemic conditions have a detrimental effect on many tissues and cell types, especially the retinal vascular cells including early loss of pericytes (PC). However, the mechanisms behind this selective sensitivity of retinal PC to hyperglycemia are undefined. The O-linked β-N-acetylglucosamine (O-GlcNAc) modification is elevated under hyperglycemic condition, and thus, may present an important molecular modification impacting the hyperglycemia-driven complications of diabetes. We have recently demonstrated that the level of O-GlcNAc modification in response to high glucose is variable in various retinal vascular cells. Retinal PC responded with the highest increase in O-GlcNAc modification compared to retinal endothelial cells and astrocytes. Here we show that these differences translated into functional changes, with an increase in apoptosis of retinal PC, not just under high glucose but also under treatment with O-GlcNAc modification inducers, PUGNAc and Thiamet-G. To gain insight into the molecular mechanisms involved, we have used click-It chemistry and LC-MS analysis and identified 431 target proteins of O-GlcNAc modification in retinal PC using an alkynyl-modified GlcNAc analog (GlcNAlk). Among the O-GlcNAc target proteins identified here 115 of them were not previously reported to be target of O-GlcNAc modification. We have identified at least 34 of these proteins with important roles in various aspects of cell death processes. Our results indicated that increased O-GlcNAc modification of p53 was associated with an increase in its protein levels in retinal PC. Together our results suggest that post-translational O-GlcNAc modification of p53 and its increased levels may contribute to selective early loss of PC during diabetes. Thus, modulation of O-GlcNAc modification may provide a novel treatment strategy to prevent the initiation and progression of DR.

## Introduction

The prevalence of diabetes mellitus and number of people that suffer from diabetes-related complications continues to rise worldwide [1]. Diabetes predominantly affects the microvascular circulation resulting in a range of unique vascular changes, which are tissue specific [2], [3]. Hyperglycemia is the primary cause of diabetes complications, including diabetic retinopathy (DR). Diabetic retinopathy is the leading cause of vision loss in many developed countries [2]. Hyperglycemia-linked pathways, including retinal ischemia and increased vascular permeability, are augmented by hypertension, and are common pathways underlying the development of vision-threatening conditions in DR [4]. Visual loss primarily occurs from either proliferation of new retinal vessels (proliferative diabetic retinopathy) or from increased permeability of retinal vessels (diabetic macular edema) [5]. The pathogenesis of DR is multifactorial and affects all cell types in the retina. The selective degeneration of retinal pericytes (PC) is an early diabetic retinal vascular change. Retinal PC loss progresses over time, which includes endothelial cell loss, resulting in the formation of acellular capillaries. In the late stages of DR, ischemia-induced pathologic growth of new blood vessels causes catastrophic loss of vision [5]. The precise early molecular and cellular changes, which occur under hyperglycemic condition in the retinal vasculature, remain poorly understood.

The O-linked β-N-acetylglucosamine (O-GlcNAc) modification is an important target of hyperglycemia and perhaps the pathogenesis of DR. O-GlcNAc modification is one of the most common posttranslational modifications, involving a wide-range of proteins including cytoplasmic, mitochondrial and nuclear. This unique and dynamic form of glycosylation occurs by the attachment of O-GlcNAc on the hydroxyl group of serine and/or threonine residues, similar to phosphorylation. The end product of hexosamine biosynthetic pathway (HBP), uridine diphosphate N-acetylglucosamine (UDP-GlcNAc), is used for O-GlcNAc modification of proteins [6]. The HBP shares its first two steps with glycolysis; first, hexokinase phosphorylates glucose to produce glucose 6-phosphate, which is converted into fructose 6-phosphate. The majority of fructose-6-phosphate is channeled to glycolysis, 2–3% of it goes to the HBP. This pathway begins with the conversion of fructose-6-phosphate into glucosamine 6-phosphate by the rate-limiting enzyme, glutamine fructose-6-phosphate aminotransferase (GFAT), followed by the acetylation of gluocsamine-6-phosphate to N-acetyl-glucosamine-6-phosphate (GlcNAc-6-P). Next, are the two reversible reactions: the conversion of GlcNAc-6-P to GlcNAc-1-P, and formation of UDP-GlcNAc by UDP-GlcNAc pyro-phosphorylase. This high-energy molecule serves as the monosaccharide donor for the post-translational modification by O-GlcNAc transferase (OGT). O-GlcNAcase (OGA) removes O-GlcNAc modification from proteins [7].

Hyperglycemia may accelerate HBP, and several studies suggest that altered O-GlcNAcylation may be involved in insulin resistance and the pathogenesis of diabetes complications [8], [9], [10]. However, very little is known about how this modification, and its protein targets, are altered in the retinal vascular cells, and contribute to the pathogenesis of DR. In our recent work, we showed that the level of O-GlcNAcylation varies both at the basal level and under high glucose conditions in retinal vascular cells [11]. One of the earliest vascular changes during the pathogenesis of DR is loss of retinal PC [12]. Retinal PC respond to high glucose with a significant increase in O-GlcNAc modification compared to both retinal endothelial cells (EC) and astrocytes (AC). Furthermore, high glucose and O-GlcNAc modification increasing agents (Thiamet-G and PUGNAc) attenuated the migratory activities of retinal PC but not retinal EC or AC [11]. Here we also found that high glucose and elevated O-GlcNAc modification increased apoptosis of retinal PC, but not retinal EC or AC. By using alkynyl-modified GlcNAc analog (GlcNAlk), in combination with a biotin affinity tag, we have identified 431 O-GlcNAc modified target proteins in retinal PC, 115 of which were not previously reported. Functional categorization of target proteins indicated that 34 are involved in cell death related pathways. Increased O-GlcNAc modification of these proteins and their altered functions could be responsible for the selective PC loss under hyperglycemic conditions.

## Materials and Methods

### Isolation and Culture of Primary Retinal Vascular Cells

Retinal vascular cells including retinal EC, PC, and AC were isolated from 4-weeks old C57BL/6-Immorto mice and cultured as we have previously described [12], [13], [14], [15]. Multiple isolations of these cells are available in the laboratory and their identity has been confirmed by staining for cell specific markers and analyzed by FACScan caliber flow cytometer.

All experiments were carried out in accordance with the Association for Research in Vision and Ophthalmology Statement for the Use of Animals in Ophthalmic and Vision Research and were approved by the Institutional Animal Care and Use Committee of the University of Wisconsin School of Medicine and Public Health.

### Trypan Blue Exclusion Test of Cell Viability

Cells grown under 5 or 25 mM glucose for 5 days and 100 nM Thiamet-G (Cayman, Ann Arbor, MI), 50 µM PUGNAc (Sigma, St. Louis, MO), 50 µM DON (Sigma) or 2.5 mM Alloxan (Sigma) added to medium of treatment groups 1 day before harvesting them. Next, cells trypsinized and mixed 1∶1 with 0.4% trypan blue solution (Sigma). Viable cells exclude trypan blue, while dead cells stain blue due to trypan blue uptake counted on hemocytometer. Experiments were repeated at least three times for each condition.

### Cell Proliferation Assay

Cells were plated in 96 well plates and incubated with or without inhibitors as described above. The proliferation values of live cells was determined using the CellTiter 96 AQueous Non-Radioactive Cell Proliferation Assay kit (Promega, Madison, WI).

### TUNEL Assay

Cells were seeded on coverslips and cultured under indicated conditions. Following incubation, the cells were fixed using 4% paraformaldehyde in PBS followed by a permeabilization with 0.25% TritonX-100. Next, cells stained with a terminal-deoxynucleotidyl transferase-dUTP nick end-labeling (TUNEL) assay (Click-iT TUNEL Alexa Fluor 594 Imaging Assay; Invitrogen, Carlsbad, CA) to identify cells with fragmented DNA. Nuclei were counterstained with 4′,6- Diamidino-2-phenylindole (DAPI; Vector Laboratories, Burlingame, CA). Fluorescence signals were detected with a Zeizz Axiophot and results were recorded with an Axiocam HRm digital camera. For each slide 10 images (counting ∼1000 cells) were obtained from randomly selected fields and analyzed.

### Ac_4_GlcNAlk Synthesis

Ac_4_GlcNAlk was synthesized according to literature procedures [16], [17].

### Azido-Azo-Biotin Synthesis

Azido-azo-biotin was synthesized according to literature procedure [18].

### Biotin Enrichment

This procedure was adapted from Zaro et al [17]. Retinal pericyte pellets labeled with Ac_4_GlcNAlk (200 µM) or DMSO for 16 h under low glucose conditions were resuspended in 200 µL H_2_O, 60 µL PMSF in H_2_O (250 mM), and 500 µL 0.05% SDS buffer (0.05% SDS, 10 mM TEA pH 7.4, 150 mM NaCl) with Complete Mini protease inhibitor cocktail (Roche Biosciences). To the resuspension was added 8 µL Benzonase (Sigma). The cells were incubated on ice for 30 min after which cells were lysed with 2000 µL 4% SDS buffer (4% SDS, 150 mM NaCl, 50 mM TEA pH 7.4). Following a brief sonication in a bath sonicator, the insoluble fraction was pelleted by centrifugation (10 min, 20,000×g at 15°C). Protein concentration of the soluble fraction was normalized by BCA assay (Pierce, ThermoScientific, Chicago, IL) to 1 mg/mL (10 mg total cell lysate).

Newly made click chemistry reagents were added to each sample [azido-azo-biotin tag (100 µM, 5 mM stock solution in DMSO); tris(2-carboxyethyl)phosphine hydrochloride (TCEP) (1 mM, 50 mM freshly prepared stock solution in water); tris[(1-benzyl-1-H-1,2,3-triazol-4-yl)methyl]amine (TBTA) (100 µM, 10 mM stock solution in DMSO); CuSO_4_•5H_2_O (1 mM, 50 mM freshly prepared stock solution in water)] for a total reaction volume of 10 mL. The reaction was allowed to proceed for 75 minutes. Ice-cold methanol (4 volumes) was then added to the reaction and precipitation proceeded at −80°C for 2 h. Precipitated proteins were centrifuged at 5,200×g for 30 min at 0°C and washed 3× with 40 mL ice-cold methanol, with resuspension of the pellet each time. The pellet was then dried for 1 h. The protein pellets were then resuspended in 4 mL of resuspension buffer (6 M urea, 2 M thiourea, 10 mM HEPES pH 8.0) by bath sonication. The captured proteins were incubated with freshly-made 1 mM dithiothreitol (100 mM stock solution; Sigma) for 40 min to reduce cystienes. Further incubation with freshly prepared 5.5 mM iodoacetamide (550 mM stock solution; Sigma) for 30 min in the dark capped reactive cystienes. Streptavidin beads (250 µL; ThermoScientific) were washed 2× with PBS (1 mL) and 1× with resuspension buffer (1 mL) before being added to proteins. Proteins were incubated on a rotator for 2 h, washed 2× with resuspension buffer, 2× with PBS and 2× with 1% SDS in PBS (10 mL per wash, 2,000×g, 2 min). Samples were transferred to 2 mL dolphin-nosed tubes.

To cleave proteins from the beads, beads were incubated in 250 µL of sodium dithionite solution (1% SDS, 25 mM sodium dithionite) for 30 min at room temperature. Following centrifugation (2,000×g, 2 min), the eluent was collected. The elution step repeated and the combined eluent precipitated with 2 mL of ice-cold methanol. The precipitated proteins were collected by centrifugation (10 min, 10,000×g at 0°C), dried and resuspended in a minimal amount for 4% SDS buffer (20 µL). 2× SDS-Free loading buffer (20% glycerol, 0.2% bromophenol blue, 1.4% β-mercaptoethanol) was then added to the samples, and the samples were boiled for 10 min. The majority of this resuspended solution, 90%, was loaded onto SDS-PAGE for in-gel trypsin digestion, while the remaining sample was loaded onto another SDS-PAGE for validation of protein candidates by Western blot analysis.

### LC-MS Analysis

Each lane of the SDS-PAGE gel was sliced into 10 fractions, and each excised gel slice was placed in a microcentrifuge tube. The gel slices were washed 2× with 50 mM ammonium bicarbonate (ABC, 300 µl, 15 min), destained 2× with a 1∶1 solution of 50 mM ABC/acetonitrile for 30 min, and then dehydrated in 100% acetonitrile. After drying the gel pieces in a SpeedVac, gel pieces were rehydrated in a trypsin solution (2 µg of trypsin per gel slice) and incubated at 37°C in a water bath for 18 h. The peptides were eluted in 50% acetonitrile in H_2_O with 0.1% TFA (200 µl, 2×), and SpeedVac dried. Samples were then subjected to nano-HPLC/MS/MS analysis (Thermo LTQ-Orbitrap in the Proteomic Resource Center at Rockefeller University).

LC-MS analysis was performed with a Dionex 3000 nano-HPLC coupled to an LTQ-Orbitrap ion trap mass spectrometer (ThermoFisher). Peptides were pressure-loaded onto a custom-made 75-µm–diameter, 15-cm C18 reverse-phase column and separated with a gradient running from 95% buffer A (HPLC water with 0.1% (v/v) formic acid) and 5% buffer B (HPLC-grade CH3CN with 0.1% (v/v) formic acid) to 55% B over 30 min, next ramping to 95% B over 10 min and holding at 95% (v/v) B for 10 min. One full MS scan (300–2000 MW) was followed by three data-dependent scans of the nth most intense ions with dynamic exclusion enabled. Peptides were identified using SEQUEST version 28 and were searched against the mouse International Protein Index (IPI) protein sequence database v3.45. Scaffold software (Proteome Software) was used to compile data.

### Western Blot Analysis

Cell lysates were separated by electrophoresis on precast Tris-Glycin 4–20% gradient gels (Invitrogen) and transferred to the Protran nitrocellulose membrane (VWR, Chicago, IL). The membranes were incubated with an anti-p53 [FL-393] (Santa Cruz Biotechnology, Santa Cruz, CA), anti-HSP90 (Cell Signaling, Boston, MA), anti-Galectin-1 [EPR3205] (Abcam, Cambridge, MA) and anti-β actin [BA3R] (Thermo). The blots were washed, incubated with appropriate secondary antibody, and developed using enhanced chemiluminescence reagents (ECL; Thermo Fisher).

### Immunoprecipitation

Immunoprecipitation of p53 protein was carried out using the anti-p53 antibody conjugated agarose beads (Santa Cruz Biotechnology). Lysate (equivalent to 500 µg total protein) was incubated with 10 µl of anti-p53 antibody conjugated agarose beads for over night at 4°C with gentle shaking. After washing the resin three times with lysis buffer, the beads were incubated with 40 µl of SDS±PAGE loading buffer for 1 min and then centrifuged at 2,000×g for 1 min to collect eluted antigen. The eluent was run on Tris-Glycin 4–20% gradient gels (Invitrogen), analyzed by western blotting as described above.

### Statistical Analysis

Experiments were repeated at least 3 times. Quantitative results were expressed as mean±SEM. ANOVA and t tests were used for statistical analysis, with P<0.05 considered significant.

## Results

### Hyperglycemia and Elevated O-GlcNAc Modification Increases Apoptosis of Retinal PC, but not Retinal EC and AC

**Figure 1:**
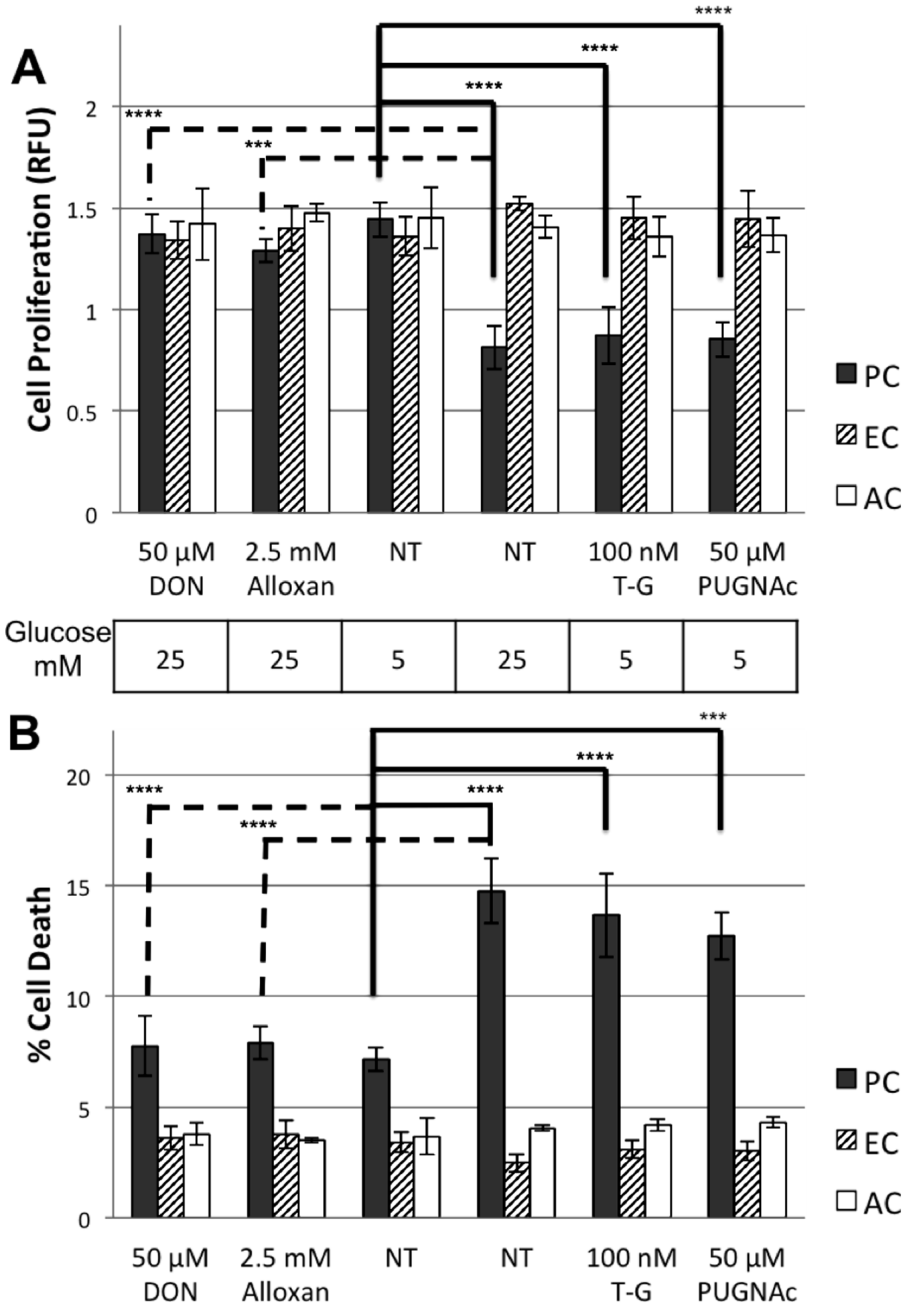
Effects of high glucose and O-GlcNAc modification inducers on retinal vascular cell death and proliferation. Cells were assayed for cell death (A) and cell proliferation (B) under varying glucose concentration, with or without O-GlcNAcylation inhibitors, Don and Alloxan, and with or without O-GlcNAcylation inducers, Thiamet G (T–G) and PUGNAc. Cell viability was assessed by counting trypan blue-positive cells. Proliferation rates were determined by a MTS-based assay. High glucose conditions, or low glucose with O-GlcNAcylation inducers, significantly increased PC death (A) as well as decreased cell proliferation (B) compared to both EC and AC. Conversely, O-GlcNAcylation inhibitors neutralized the negative effects of high glucose on retinal PC. Mean ± SEM; ***(p≤0.01), and ****(p≤0.001) significantly different from 5 mM glucose control.

We have examined the effect of high glucose conditions on proliferation and apoptosis of retinal PC, EC and AC. Exposure to 25 mM glucose resulted in decreased viability of retinal PC, which was not observed in EC and AC (Fig. 1A). Moreover, agents known to increase O-GlcNAc modification, Thiamet-G and PUGNAc, decreased the viability of retinal PC cultured under normal glucose conditions (5 mM). Conversely, using agents capable of reducing O-GlcNAc modifications, DON and Alloxan, prevented the negative effect of high glucose on cell viability in retinal PC (Fig. 1A).

**Figure 2:**
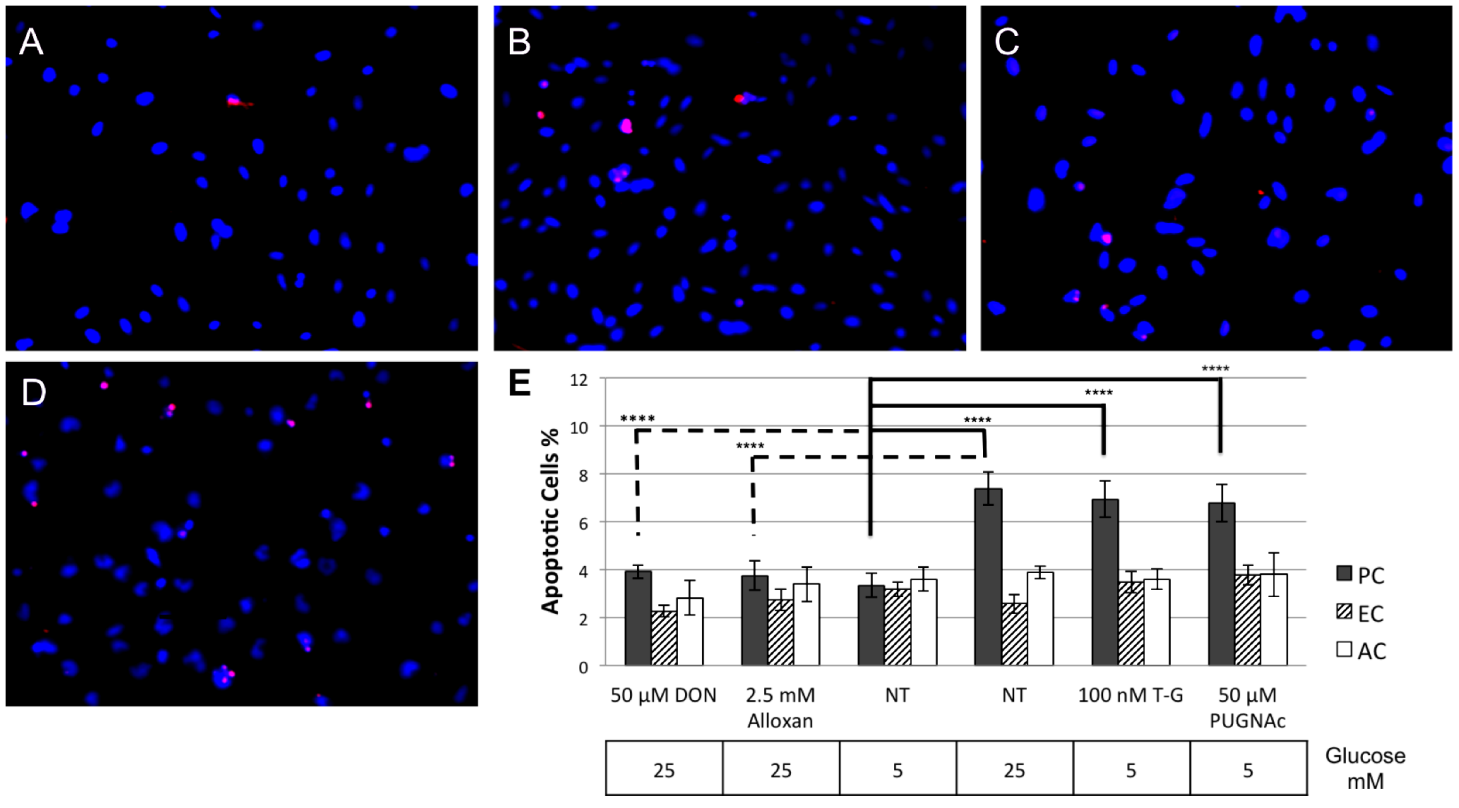
Effects of high glucose and O-GlcNAc modification inducers on apoptosis of retinal vascular cells. TUNEL staining was used to detect cell apoptosis (red). The nuclei were counterstained with DAPI (blue). Violet color represents TUNEL-positive nuclei on merged photos. (A): represents retinal PC grown in 5 mM glucose medium, (B): in 25 mM glucose medium, (C): treatment with 100 nM Thiamet-G for 1 day in 5 mM glucose medium, (D): positive control, cells treated with 1 µM staurosporine (STP) for 6 h. These images are representative of images evaluated at least 1000 cells for each condition with 3 replicates (original magnification x200). (E); Bar graphs quantify apoptosis, which is expressed as percentage of apoptotic cells for each condition. Data are presented as mean ± SEM (n = 3). Mean ± SEM; ****(p≤0.001) significantly different from 5 mM glucose control.

The decreased cell viability with high glucose was associated with increased rate of cell death in retinal PC, but not in retinal EC and AC (Fig. 1B). Thiamet-G and PUGNAc induced cell death in retinal PC cultured under normal glucose conditions (5 mM). DON and Alloxan prevented the high glucose mediated cell death in retinal PC (Fig. 1A). Furthermore, we detected a 2-fold increase in apoptosis of retinal PC under high glucose condition (25 mM glucose) compared with normal glucose (5 mM glucose) (Fig. 2). Together, these results, along with O-GlcNAc modification level profiles [11] suggest that the elevation of O-GlcNAc modification is responsible for the effects of hyperglycemia on retinal PC vitality.

### Identification of O-GlcNAcylation Target Proteins in Retinal Pericytes

To delineate the pathways in which O-GlcNAc modification contribute to retinal PC dysfunction require identifying of the target proteins. Reliable purification techniques have thus far limited the identification of O-GlcNAcylated proteins. However, a method developed by our group has improved the purification of O-GlcNAcylated proteins by utilizing chemical reporters and click-It chemistry [17]. We performed a large-scale enrichment from retinal PC using an alkynyl-modified GlcNAc analog (GlcNAlk) as a chemical handle. Identified proteins were compiled and categorized into high- and medium-confidence lists, based on the number of assigned spectra and the fold increase above control (Table S1 and S2 in File S1). Using this technology, we have identified 431 proteins by GlcNAlk labeling; representing proteins with diverse cellular functions, and over 115 of which are considered novel targets as they have not previously been reported as targets of O-GlcNAc modification.

**Figure 3:**
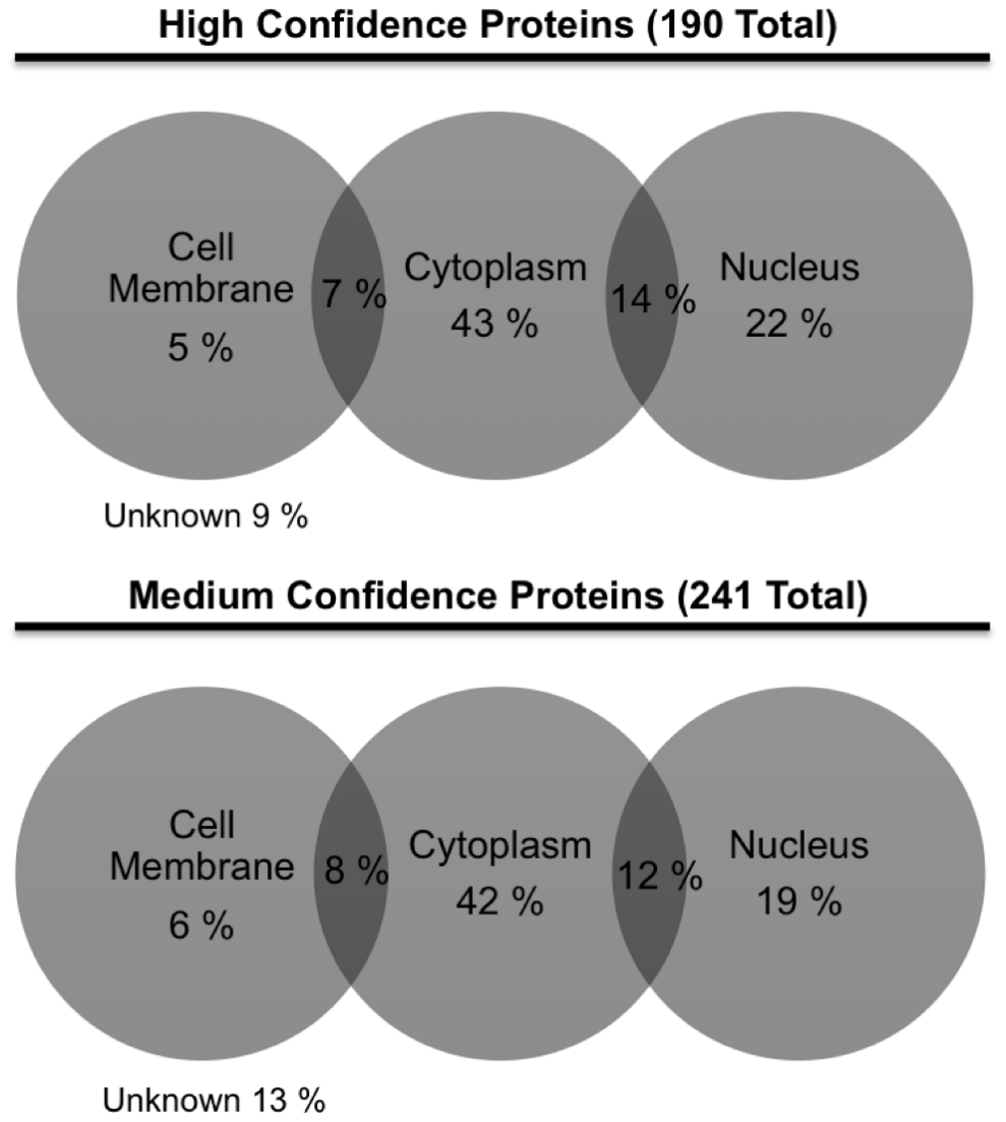
Subcellular localization of identified O-GlcNAc proteins in retinal PC. Proteins have dual localization, indicated in overlapping areas. The complete list of identified proteins provided in Table S1 and Table S2 in File S1.

The GO term analysis for subcellular localization of the identified proteins (high confidence group) resulted with 43% cytoplasmic, 22% nuclear and 5% cell membrane localization (Figure 3). We also determined that a group of the identified proteins have dual localization: 14% cytoplasm+nucleus and 7% cytoplasm+cell membrane. Subcellular localization of medium confidence group has a similar distribution: 42% cytoplasm, 19% nucleus, 6% cell membrane, 12% cytoplasm+nucleus, 8% cytoplasm+cell membrane (Figure 3).

### Functional Characterization of O-GlcNAc Modified Proteins

**Figure 4:**
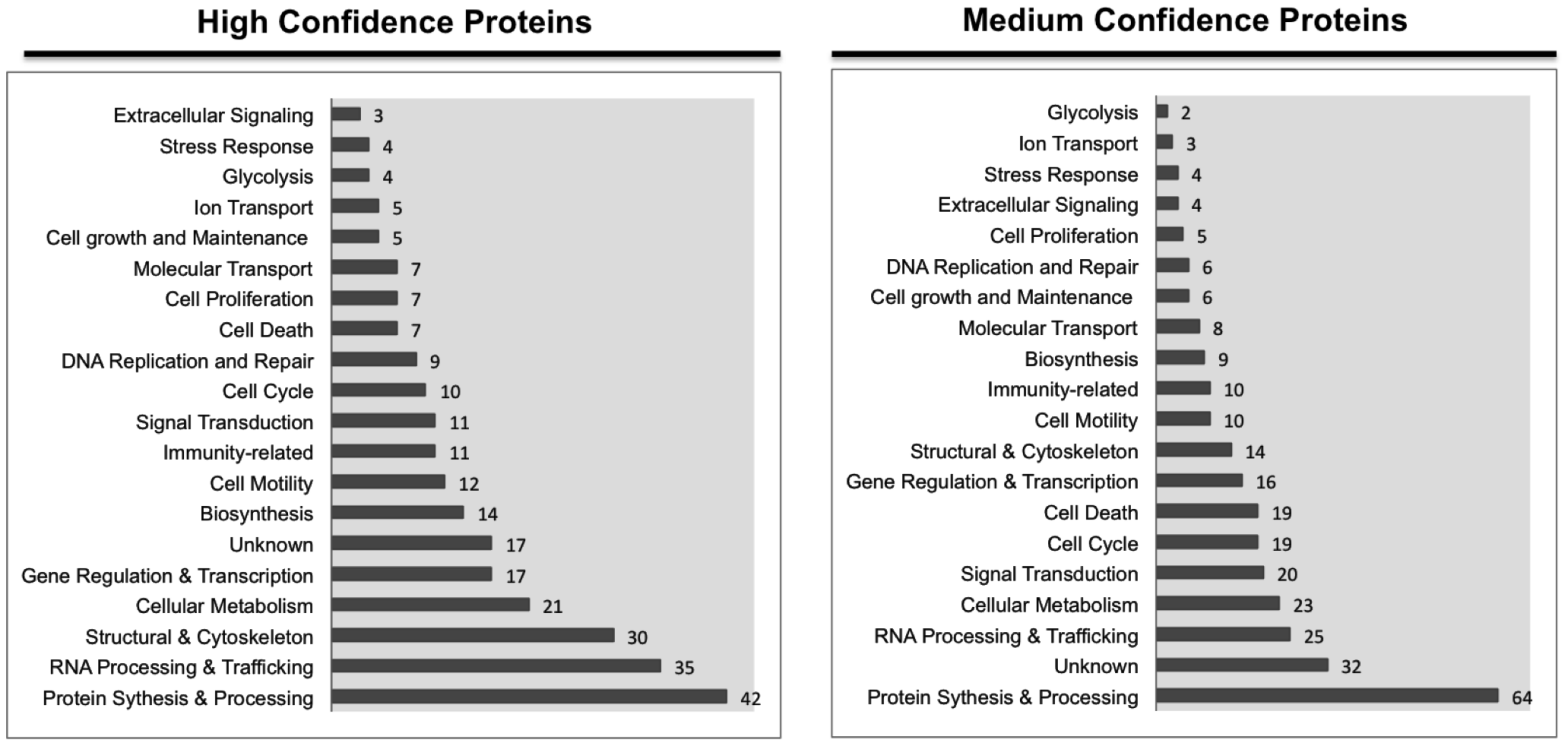
Functional categories of identified O-GlcNAc proteins in retinal PC. Multifunctional proteins are included in more than one functional category. The complete list of identified proteins provided in Table S1 and Table S2 in File S1.

**Figure 5:**
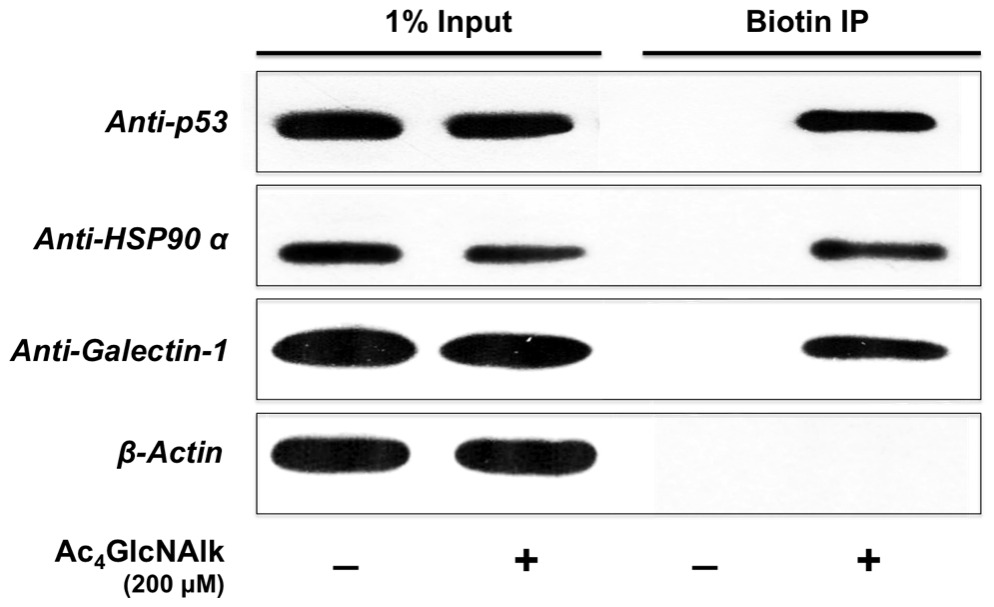
Confirmation of identified proteins by Western blot analysis. O-GlcNAlk-modified proteins were enriched from retinal PC treated with Ac4GlcNAlk (200 µM) using azido-azo-biotin and analyzed by Western blotting. 1% of lysates (input) loaded on gel to confirm the existence of proteins in starting material and to indicate no change in expression levels of proteins after Ac4GlcNAlk treatment (Lane 1 and 2). Ac4GlcNAlk-biotin incorporated proteins precipitated using Streptavidin beads (Lane 4). Control cells are not treated with Ac4GlcNAlk (Lane 1 and 3). Membranes are blotted with anti-p53, anti-HSP90 and anti-Galectin-1 antibodies as representative of identified proteins.

The identified proteins were functionally analyzed and grouped by using Uniport database. Functional characterization of identified proteins indicated that O-GlcNAc modified proteins are involved in a broad range of cellular pathways and biological processes as have been shown in previous proteomic studies (Figure 4) [17], [19]. To confirm our purification method, we performed Western blot analysis of a subset of GlcNAlk enriched proteome using antibodies against these proteins (Figure 5). Taken together, the high numbers of O-GlcNAc modified proteins involved in protein synthesis, gene regulation, cellular metabolism and other pathways reflect the potential effects of increased O-GlcNAc modification on retinal PC under hyperglycemia.

### Proteins that are Involved in the Cell Death Processes are among the Identified O-GlcNAcylation Targets

Initially, we focused our attention on O-GlcNAc modified proteins, which are specifically involved in cell death pathways, because of the established sensitivity of retinal PC to hyperglycemia. We determined that at least 34 of the identified proteins in our proteomic list are involved in the cell death processes. The possible roles of these identified proteins in cell death are listed in Table 1. This data provides a number of target proteins and their potential involvement in the process of early retinal PC loss under hyperglycemia. Among these proteins, p53 is a well-known and widely studied protein, and previous studies have shown a link to p53 degradation via O-GlcNAc modifications [20]. Besides p53, this list includes a number of proteins that have important roles in cell cycle and death, such as BAX, heat shock proteins (HSP), peroxiredoxins, 14-3-3 protein zeta/delta, active regulator of SIRT1 and Bag3 (Table 1). The knowledge about the effects of O-GlcNAc modification on these proteins is very limited. It has, however, been reported that O-GlcNAc regulates both the rates and extent of the stress-induced induction of HSPs [21]. PKC activation by a reduction in 14-3-3 zeta in the retina suggested a cause of visual dysfunction during diabetes [22]. An increase in apoptotic cells, as well as elevated protein expression of Bax were reported in the retina of diabetic rats [23]. It has also been shown that retinal SIRT1 activity is significantly lower in the diabetic mice [24]. In this manner, our results provide a list of proteins regulated by O-GlcNAc modification in retinal PC with important role in cell survival. Functional or expressional alterations in some of these proteins have been already reported in diabetic retina, however the mechanisms that cause these alterations were undefined. Furthermore, some of the proteins identified here are novel targets whose role in the pathogenesis of DR needs further study.

**Table 1:** The list of O-GlcNAc modified proteins involved in cellular death processes.

Protein Name	Gene	
14-3-3 protein zeta/delta	**Ywhaz**	Involves heterodimerization of Raf kinases that initiate the activation of the extracellular signal-regulated kinase (ERK) cascade, which, in turn, promotes proliferative and survival signaling [26]. Suppress apoptosis through interactions with BCL-2 antagonist of cell death (BAD), BCL-2 interacting mediator of cell death (BIM) and BCL-2 associated x protein (BAX), and through interactions with proteins that transmit apoptotic signals, including the stress-responsive kinase ASK1 (MEKK5) and the forkhead box O1 (FOXO) transcription factors [26], [27].
Active regulator of SIRT1	**Rps19bp1**	Directly binds SIRT1 to enhance SIRT1-mediated deacetylation of p53 in vitro and in vivo, which inhibits p53-mediated transcriptional activity [28].
	Aros	
Aminoacyl tRNAsynthase complex-interactingmultifunctional protein 1	**Aimp1**	Inhibits endothelial proliferation via JNK-dependent apoptosis as its level is increased [29]. A potent chemoattractant for monocytes, associated with the clearing sites of apoptotic cell debris by phagocytosis. Induce apoptosis in cultured ECs especially when ECs were exposed to hypoxia [30].
	Emap2	
	Scye1	
Anamorsin	**Ciapin1**	Anti-apoptotic. Inhibition of CIAPIN1 promotes apoptosis of vascular smooth muscle cells (VSMCs) by regulating Bcl-2 and Bax [31].
Apoptosis regulator BAX	**Bax**	Plays a central role in the mitochondria-dependent apoptotic pathway. Following a death signal, the protein is translocated to the outer mitochondrial membrane, where it promotes a permeabilization that favors the release of different apoptogenic factors, such as cytochrome c [32]
Aquaporin-1	**Aqp1**	Controls the water loss in the regulation of the apoptotic volume decrease (AVD) and, therefore, the beginning of the process of cell death [33].
BAG family molecularchaperone regulator 3	**Bag3**	Anti-apoptotic. Protects IKK-γ from proteasome delivery and this result in sustained NF-kB activation and cell survival [34], [35]. Retains BAX protein in the cytosol, preventing its mitochondrial translocation [35], [36].
	Bis	
Catenin alpha-1	**Ctnna1**	Loss of α-catenin decreases or increases apoptosis appears to be dependent on the cellular context [37].
	Catna1	
CDKN2A-interacting protein	**Cdkn2aip**	May activate p53 function by ARF-mediated or ARF-independent mechanisms [38], [39]. A stabilizer and activator of p53, and suppressor of p53 antagonists [40].
	Carf	
Cellular tumor antigen p53	**Trp53**	Well-known inducer of apoptosis by transcription dependent or independent mechanisms [41], [42].
	Tp53	
	P53	
DNA topoisomerase 2-alpha	**Top2a**	Involves the formation of condensed and fragmented chromatin associated with apoptosis. Overexpression or deregulation expression triggers apoptotic cell death [43].
Dynamin-1-like protein	**Dnm1**	Have roles in mitochondrial fission process and apoptosis progression [44].
	Drp1	
Galectin-1	**Lgals1**	Activates extracellular signal–regulated kinase-2 (ERK-2), induces the transcription factor AP-1, down-regulates the anti-apoptotic protein Bcl-2, thus a predominates the pro-apoptotic protein Bax and activates caspases [45].
Heat shock 70 kDa protein 4	**Hspa4**	A powerful anti-apoptotic protein, inhibits the TRAIL-induced assembly of the death-inducing signaling complex (DISC), stabilize Akt, inhibits BID activation [46].
Heat shock protein HSP 90-α and -β	**Hsp90aa1**	A power anti-apoptotic protein by regulating ranscription factors and kinases implicated in apoptosis, such as NF-κB, p53, Akt, Raf-1 and JNK [46].
	**Hsp90ab1**	
Heat shock protein 105 kDa	**Hsph1**	Attenuates staurosporine induced apoptosis, but overexpressed HSP105α in mouse embryonal F9 cells enhanced apoptosis in response to and HSP105 is required for caspase-3-mediated apoptosis following ER stress [47].
	Hsp105	
Histone deacetylase 6	**Hdac6**	Deacetylates Ku70, keeps it in complex with Bax, inhibits Bax-induced cell death. Deacetylates survivin and triggers its nuclear export in a mechanism that controls nuclear acetylated survivin levels and blocks its apoptotic effect [48].
Nestin	**Nes**	Degradation of nestin is shown to be a prerequisite for activation of Cdk5 and induction of apoptosis during oxidative stress [49].
Nucleophosmin	**Npm1**	Inhibits apoptosis induced by a number of factors, including c-Myc, hypoxia and UV irradiation [50].
Peroxiredoxin-1, -2 & -4	**Prdx1**	Peroxiredoxins are important in eliminating ROS from inducing cytotoxicity. However, when the peroxide levels are sufficiently high to induce hyperoxidation of Prx I, the hyperoxidized high molecular weight oligomers of Prx I have been shown to bind and activate MST1 kinase, which in turn induces apoptosis via a p53-mediated pathway [51].
	**Prdx2**	
	**Prdx4**	
Phospholipid hydroperoxideglutathione peroxidase, mitochondrial	**Gpx4**	Counteracted the 12,15-lipoxygenase (LOX) and apoptosis inducing factor (AIF) mediated apoptosis [52].
Prelamin-A/C	**Lmna**	Caspase-6- mediated proteolysis of lamin A/C is crucial for nuclear apoptotic events such as shrinkage, disassembly of nuclear membrane and formation of apoptotic bodies [53].
	Lmn1	
Probable ATP-dependentRNA helicase DDX17	**Ddx17**	Interact with Ddx5 and coactivate p53-dependent transcription [54].
	p72	
Programmed cell death 6-interacting protein	**Pdcd6ip**	Acts upstream of caspase 9 activation following cytosolic calcium elevation [55]. Interaction with ALG-2 is important in cell death regulated by TNFα receptor-1 [56].
	Aip1	
	Alix	
Reticulon-3	**Rtn3**	Directly involved in the endoplasmic reticulum-constituents trafficking events through dually acting as an essential and important ER-stress sensor, and a trigger for the Bcl-2 translocation [57].
Receptor-interactingserine/threonine-protein kinase 2	**Ripk2**	Involves in the regulation of apoptosis induced by the CD95 receptor pathway [58].
	RICK	
Ribosomal protein S6kinase alpha-3	**Rps6ka3**	Promotes cell survival by increasing CREB-dependent transcription of survival-promoting genes, including Bcl-2, Bcl-xL and Mcl1 [59].
	RSK3	
RNA-binding protein 25	**Rbm25**	Activates proapoptotic Bcl-x_s_ 5′ ss via its interaction with the exonic splicing enhancer, CGGGCA [60].
Serine/threonine-proteinphosphatase 2A 65 kDaregulatory subunit A alpha isoform	**Ppp2r1a**	Acts as a negative regulator for the Akt pathway. Phosphorylation of BAD suppresses, and its dephosphorylation by PP2A promotes pro-apoptotic activity [61]. Positively regulates the pro-apoptotic activity of FOXO_1_ [62].
Translationally-controlledtumor protein	**Tpt1**	P53-dependent induction of Tpt1 is able to reduce oxidative stress, minimize apoptosis, and promote cell survival in response to H_2_O_2_ challenge [63].
Ubiquitin carboxyl-terminal hydrolase 10	**Usp10**	Stabilizes p53; deubiquitinates p53 thereby allowing its re-entry into the nucleus [64].
	Kiaa0190	
	Ode-1	
	Uchrp	

### Increased p53 Levels in Retinal PC under High Glucose Conditions

**Figure 6:**
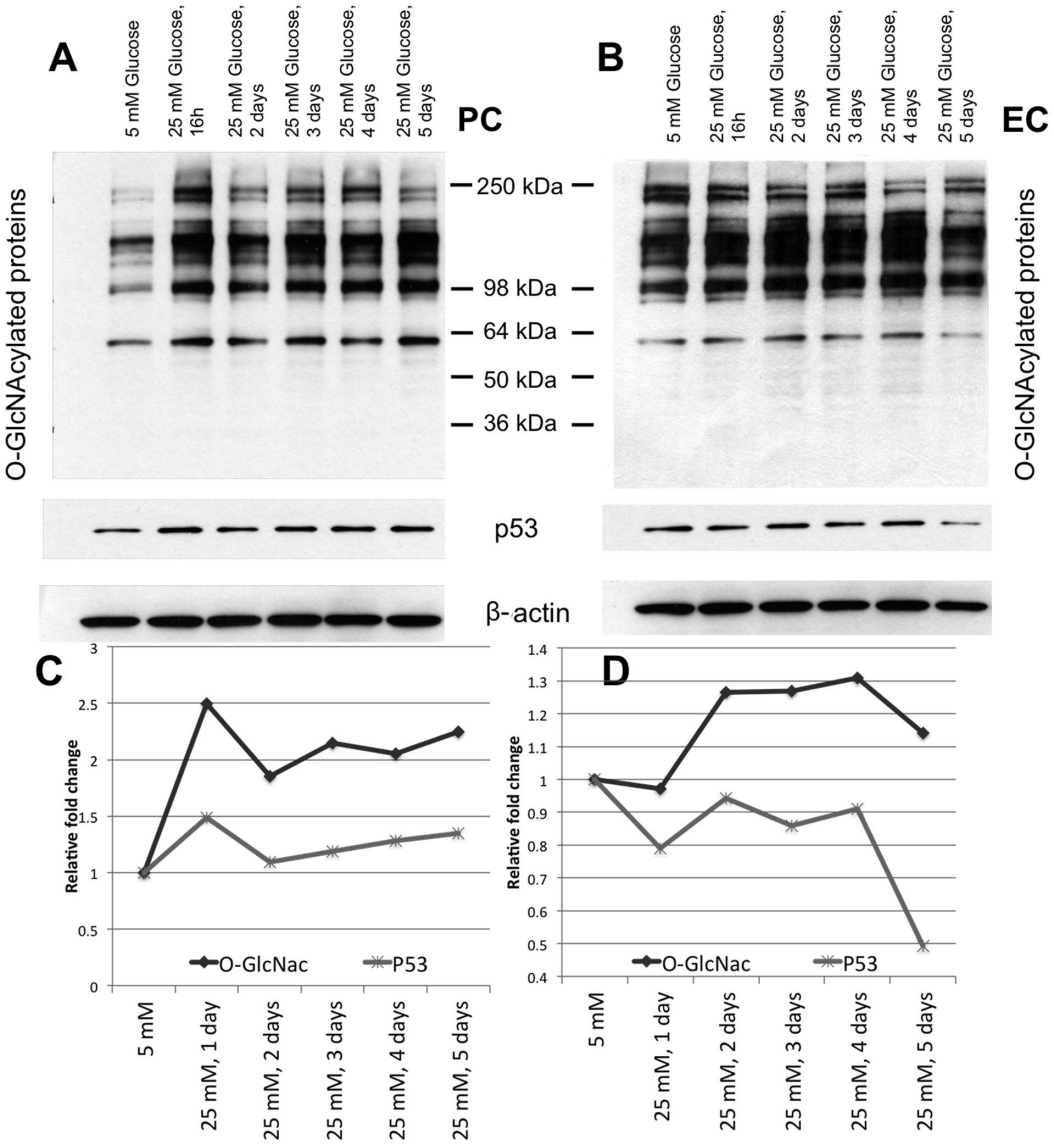
Alterations in the levels of total O-GlcNAc modified proteins and p53. Retinal PC (A and C) and EC (B and D) under high glucose. Protein lysates (50 µg) were analyzed by Western blot analysis for O-GlcNAcylated proteins and p53 under 5 mM (1^st^ lane) and 25 mM glucose respectively for 1 day, 2, 3, 4 and 5 days. The β-actin expression was assessed as a loading control and used for normalization and quantification. Please note the increase in O-GlcNAc and p53 levels under high glucose conditions in retinal PC but not EC.

We detected an elevation of p53 protein level in retinal PC cultured under high glucose (25 mM) compared with normal glucose (5 mM) conditions. The protein expression profile of p53 showed an elevation, which paralleled the increased O-GlcNAc modification level in retinal PC under 25 mM glucose for different days (Fig. 6A & C). The p53 RNA expression levels, as assessed by quantitative PCR, were not altered in retinal vascular cell (not shown). Finally, we did not detect an increase in p53 protein levels in retinal EC (Fig. 6B & D) and retinal AC (not shown) under high glucose conditions, corresponding with the limited alterations in O-GlcNAc modification and the rate of apoptosis in these cells (Fig. 2).

**Figure 7:**
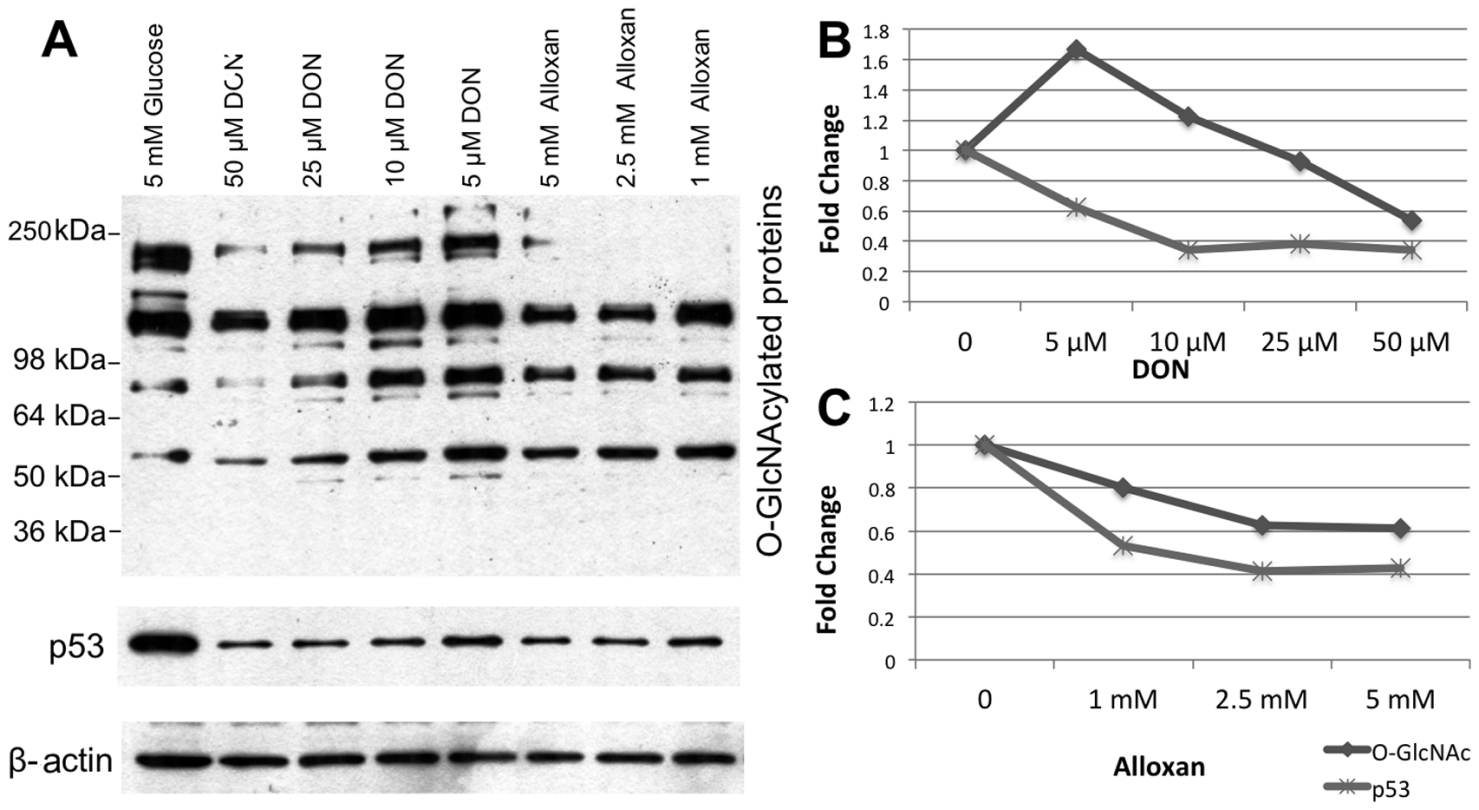
Alterations in the levels of total O-GlcNAc modified proteins and p53 in retinal PC incubated with DON and Alloxan for 16 **h.** Protein lysates (50 µg) from retinal PC were analyzed by Western blot analysis for O-GlcNAcylated proteins and p53 (A). All treatments applied with 5 mM glucose in medium. The β-actin expression was assessed as a loading control and used for normalization and quantification (B and C). Please note a decrease in level of O-GlcNAc modifications and p53 levels.

In order to assess whether decreased O-GlcNAc modification negatively impacted p53 expression, we applied the GFAT inhibitor DON (Fig. 7A & B) and the OGT inhibitor Alloxan (Fig. 7A & C) under 5 mM glucose conditions in order to reduce O-GlcNAc modification, lower than basal level. Under these conditions, we found that p53 was subject to a dose-dependent decrease in protein levels following incubation with GFAT and OGT inhibitors, which again paralleled with a decrease in the levels of O-GlcNAc modification. These results further emphasize the contribution of O-GlcNAc modification to modulation of p53 protein levels in retinal PC.

**Figure 8:**
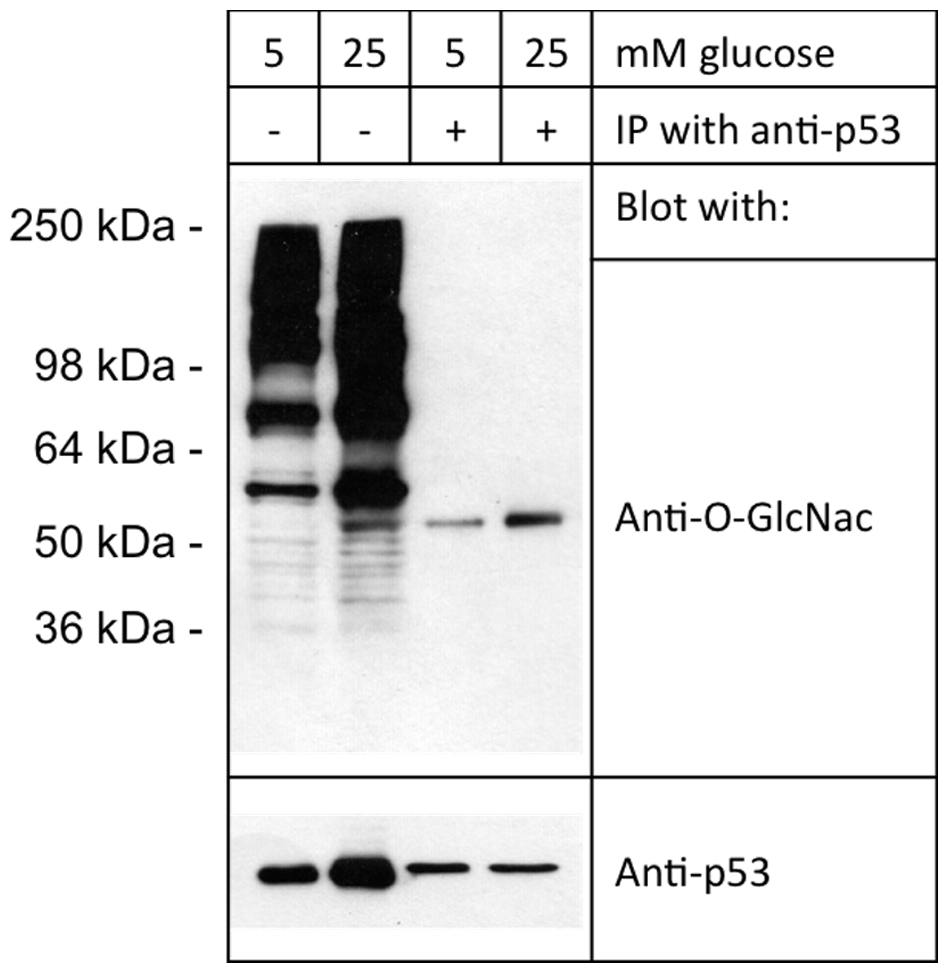
Increased O-GlcNAc modification of p53 in retinal PC under different glucose conditions. Same amount of p53 was precipitated from retinal PC lysates (equivalent to 500 µg total protein) with anti-p53 antibody conjugated agarose beads. Line 1 and 2; beginning materials for 5 and 25 mM glucose conditions. Line 3 and 4; precipitated p53. Please note increased O-GlcNAc modified p53 under high glucose conditions.

Next, we performed an immunoprecipitation (IP) assay of p53 to specifically determine the O-GlcNAc modification of the protein by using anti-p53 conjugated agarose beads in retinal PC lysates. We precipitated the same concentration of p53 protein from PC grown in 5 mM or 25 mM glucose. Blotting with anti-O-GlcNAc antibody, we found that O-GlcNAc modified p53 level was increased in PC grown under 25 mM glucose conditions. Together, these data demonstrated that p53 levels increased in a cell-specific manner in parallel with increased O-GlcNAc modification under high glucose conditions (Fig. 8). Thus, O-GlcNAcylation of p53 resulted in its increased levels perhaps by interfering with its proteasome-mediated degradation.

## Discussion

For over 50 years hyperglycemia has been recognized as the primary cause for the majority of diabetes complications. Although the target tissues, and even target cells, of diabetes have been recognized, the detailed molecular mechanisms involved in hyperglycemia-mediated damage remain unclear. In addition, the incentive factors for which hyperglycemia targets specific tissues/cells remains poorly understood. We hypothesized that increased O-GlcNAc modification is involved in the progress of hyperglycemia driven complications and its target/tissue specificity. We and others have found that hyperglycemia induces O-GlcNAc modifications in a cell specific manner [11], [17]. Furthermore, O-GlcNAc modification affects a wide range of proteins, including transcription factors, stress factors, proteins involved in RNA and protein synthesis and processing, as well as those proteins involved in other post translational modifications [16], [17], [19].

The involvement of O-GlcNAc modification in the post-translational modifications of a wide range of proteins suggests a possible role in the regulation of many cellular pathways. The target proteins of O-GlcNAc modification, and their contribution to the pathogenesis of diabetes complications in affected tissues remain unknown. This may be due, in part, to the dynamic and unstable constitution of this modification, and to the difficulty of the purification of O-GlcNAcylated proteins. We recently described a new technique to improve the enrichment and identification of O-GlcNAc modified proteins by using GlcNAc analogs and click-it chemistry [17]. This method is very efficient for global identification of proteins, which are target of O-GlcNAc modification.

We recently showed that hyperglycemia causes an increase in O-GlcNAc modification of retinal vascular cell proteins in a cell-specific manner. In focusing on the retinal vascular cells, which are the target of hyperglycemia-driven DR, we found that retinal PC are more susceptible to the elevation in O-GlcNAc modification under high glucose conditions compared with retinal EC or AC [11]. Interestingly, PC loss is one of the earliest changes detected in the pathogenesis of DR [12]. This overlap led us to investigate the role of increased O-GlcNAc modification in early PC loss during diabetes.

Here we showed a significant increase in apoptosis of retinal PC under high glucose conditions or by treatment with O-GlcNAc increasing pharmacological agents (Figure 1 & 2). However, we did not detect any effect on death of retinal EC or AC, under high glucose conditions or by exposure to O-GlcNAc-inducing agents. Furthermore, inhibition of O-GlcNAc modification under high glucose conditions protected retinal PC from apoptosis. Thus, high glucose mediated O-GlcNAc modification in retinal PC has an adverse effect on their survival.

To gain insight into the mechanisms involved, we determined the identity of proteins, which are target of O-GlcNAc modification in retinal PC. Following enrichment of O-GlcNAcylated proteins in retinal PC using GlcNAlk as chemical handle and LC-MS/MS analysis, we identified 431 proteins (Table S1 and S2 in File S1). The identified proteins spanned a broad range of cellular localizations (Figure 3) and functions (Figure 4). We have provided a list of cell death related proteins among identified proteins, and some information regarding their potential functions (Table 1). We have initially focused on specific set of proteins that may have a role in early PC loss driven by hyperglycemia and increased O-GlcNAc modification. In this manner, we demonstrated that p53 protein level was increased, specifically in retinal PC, under high glucose conditions (Figure 6).

Recent studies have indicated that O-GlcNAc modification may slow down protein degradation by directly modulating proteasome activity, regulating the ubiquitination process, or interfering/accelerating other post-translational modifications of proteins [25]. The O-GlcNAc modification regulates the degradation of p53, Δ-Lactoferrin, Snail1, Estrogen Receptor β (ERβ), Casein kinase 2 alpha (CK2α), CREB regulated transcription coactivator 2 (CRTC2), Peroxisome proliferator-activated receptor gamma co-activator 1-alpha (PGC-1α) and BMAL1/CLOCK [25]. Phosphorylation of both serine and threonine residues on the N terminus of p53 increases its stability by decreasing its interaction with Mdm2. In contrast, phosphorylation of Thr155 promotes Mdm2 and p53 interaction, and thus, increases p53 degradation. The O-GlcNAc modification of Ser149 increases p53 stabilization by preventing phosphorylation of Thr155 [20]. Collectively, these studies and our data indicate that regulation of p53 stability by O-GlcNAc modification may regulate hyperglycemia-induced cell death in retinal PC. Further *in vitro* and *in vivo* studies delineating the alterations in function, stability and localization of O-GlcNAcylated proteins under hyperglycemia will provide significant contribution towards decoding pathways involved in loss of retinal PC and pathogenesis of DR.

## Supporting Information

File S1 **Proteins selectively identified in GlcNAlk samples by mass spectrometry.** Data was considered high confidence (Table S1) if the number of assigned spectra was at least 10-fold greater for GlcNAlk samples compared with DMSO control samples. Further, the protein must have been identified with spectral counts greater than or equal to 5. Data was considered medium confidence (Table S2) if the number of assigned spectra was at least 2 fold greater for GlcNAlk samples than DMSO control samples. Further, the protein must have been identified with spectral counts greater than or equal to 2. Bolded proteins were not identified previously as O-GlcNAc modification target.
(DOCX)

## References

[1] Rathmann W, Giani G (2004) Global prevalence of diabetes: estimates for the year 2000 and projections for 2030. Diabetes Care 27: 2568–2569; author reply 2569. 10.2337/diacare.27.10.2568 15451946

[2] Frank RN (2004) Diabetic retinopathy. N Engl J Med 350: 48–58. 14702427 10.1056/NEJMra021678

[3] Gardner TW, Antonetti DA (2007) A prize catch for diabetic retinopathy. Nat Med 13: 131–132. 17290269 10.1038/nm0207-131

[4] Cheung N, Mitchell P, Wong TY (2010) Diabetic retinopathy. Lancet 376: 124–136. 20580421 10.1016/S0140-6736(09)62124-3

[5] Aiello LP (2005) Angiogenic pathways in diabetic retinopathy. The New England journal of medicine 353: 839–841. 16120866 10.1056/NEJMe058142

[6] Ngoh GA, Jones SP (2008) New insights into metabolic signaling and cell survival: the role of beta-O-linkage of N-acetylglucosamine. J Pharmacol Exp Ther 327: 602–609. 18768779 10.1124/jpet.108.143263 PMC6545568

[7] Ngoh GA, Facundo HT, Zafir A, Jones SP (2010) O-GlcNAc signaling in the cardiovascular system. Circ Res 107: 171–185. 20651294 10.1161/CIRCRESAHA.110.224675 PMC2919351

[8] McClain DA, Lubas WA, Cooksey RC, Hazel M, Parker GJ, et al (2002) Altered glycan-dependent signaling induces insulin resistance and hyperleptinemia. Proc Natl Acad Sci U S A 99: 10695–10699. 12136128 10.1073/pnas.152346899 PMC125016

[9] Whelan SA, Lane MD, Hart GW (2008) Regulation of the O-linked beta-N-acetylglucosamine transferase by insulin signaling. J Biol Chem 283: 21411–21417. 18519567 10.1074/jbc.M800677200 PMC2490780

[10] Yang X, Ongusaha PP, Miles PD, Havstad JC, Zhang F, et al (2008) Phosphoinositide signalling links O-GlcNAc transferase to insulin resistance. Nature 451: 964–969. 18288188 10.1038/nature06668

[11] Gurel Z, Sieg KM, Shallow KD, Sorenson CM, Sheibani N (2013) Retinal O-linked N-acetylglucosamine protein modifications: implications for postnatal retinal vascularization and the pathogenesis of diabetic retinopathy. Mol Vis 19: 1047–1059. 23734074 PMC3668662

[12] Cogan DG, Toussaint D, Kuwabara T (1961) Retinal vascular patterns. IV. Diabetic retinopathy. Arch Ophthalmol 66: 366–378. 13694291 10.1001/archopht.1961.00960010368014

[13] Scheef E, Wang S, Sorenson CM, Sheibani N (2005) Isolation and characterization of murine retinal astrocytes. Mol Vis 11: 613–624. 16148882

[14] Scheef EA, Sorenson CM, Sheibani N (2009) Attenuation of proliferation and migration of retinal pericytes in the absence of thrombospondin-1. Am J Physiol Cell Physiol 296: C724–734. 19193867 10.1152/ajpcell.00409.2008 PMC2670648

[15] Su X, Sorenson CM, Sheibani N (2003) Isolation and characterization of murine retinal endothelial cells. Mol Vis 9: 171–178. 12740568

[16] Gurcel C, Vercoutter-Edouart AS, Fonbonne C, Mortuaire M, Salvador A, et al (2008) Identification of new O-GlcNAc modified proteins using a click-chemistry-based tagging. Anal Bioanal Chem 390: 2089–2097. 18369606 10.1007/s00216-008-1950-y

[17] Zaro BW, Yang YY, Hang HC, Pratt MR (2011) Chemical reporters for fluorescent detection and identification of O-GlcNAc-modified proteins reveal glycosylation of the ubiquitin ligase NEDD4–1. Proc Natl Acad Sci U S A 108: 8146–8151. 21540332 10.1073/pnas.1102458108 PMC3100932

[18] Charron G, Zhang MM, Yount JS, Wilson J, Raghavan AS, et al (2009) Robust fluorescent detection of protein fatty-acylation with chemical reporters. J Am Chem Soc 131: 4967–4975. 19281244 10.1021/ja810122f

[19] Hahne H, Sobotzki N, Nyberg T, Helm D, Borodkin VS, et al (2013) Proteome wide purification and identification of O-GlcNAc-modified proteins using click chemistry and mass spectrometry. J Proteome Res 12: 927–936. 23301498 10.1021/pr300967y PMC4946622

[20] Yang WH, Kim JE, Nam HW, Ju JW, Kim HS, et al (2006) Modification of p53 with O-linked N-acetylglucosamine regulates p53 activity and stability. Nat Cell Biol 8: 1074–1083. 16964247 10.1038/ncb1470

[21] Zachara NE, O’Donnell N, Cheung WD, Mercer JJ, Marth JD, et al (2004) Dynamic O-GlcNAc modification of nucleocytoplasmic proteins in response to stress. A survival response of mammalian cells. The Journal of biological chemistry 279: 30133–30142. 15138254 10.1074/jbc.M403773200

[22] Kim YH, Kim YS, Kang SS, Noh HS, Kim HJ, et al (2005) Expression of 14-3-3 zeta and interaction with protein kinase C in the rat retina in early diabetes. Diabetologia 48: 1411–1415. 15909155 10.1007/s00125-005-1774-7

[23] Gao XY, Kuang HY, Zou W, Liu XM, Lin HB, et al (2009) The timing of re-institution of good blood glucose control affects apoptosis and expression of Bax and Bcl-2 in the retina of diabetic rats. Molecular biology reports 36: 1977–1982. 18991018 10.1007/s11033-008-9407-0

[24] Kubota S, Ozawa Y, Kurihara T, Sasaki M, Yuki K, et al (2011) Roles of AMP-activated protein kinase in diabetes-induced retinal inflammation. Investigative ophthalmology & visual science 52: 9142–9148. 22058332 10.1167/iovs.11-8041

[25] Ruan HB, Nie Y, Yang X (2013) Regulation of protein degradation by O-GlcNAcylation: crosstalk with ubiquitination. Mol Cell Proteomics. 10.1074/mcp.R113.029751 PMC3861702 23824911

[26] Morrison DK (2009) The 14-3-3 proteins: integrators of diverse signaling cues that impact cell fate and cancer development. Trends Cell Biol 19: 16–23. 19027299 10.1016/j.tcb.2008.10.003 PMC3073487

[27] Porter GW, Khuri FR, Fu H (2006) Dynamic 14-3-3/client protein interactions integrate survival and apoptotic pathways. Semin Cancer Biol 16: 193–202. 16697216 10.1016/j.semcancer.2006.03.003

[28] Kim EJ, Kho JH, Kang MR, Um SJ (2007) Active regulator of SIRT1 cooperates with SIRT1 and facilitates suppression of p53 activity. Mol Cell 28: 277–290. 17964266 10.1016/j.molcel.2007.08.030

[29] Burastero SE, Fabbri M (2012) Aminoacyl-tRNA synthetase-interacting multifunctional protein-1 (AIMP1): the member of a molecular hub with unexpected functions, including CD4 T cell homeostasis. Clin Immunol 143: 207–209. 22542741 10.1016/j.clim.2012.03.006

[30] Berger AC, Tang G, Alexander HR, Libutti SK (2000) Endothelial monocyte-activating polypeptide II, a tumor-derived cytokine that plays an important role in inflammation, apoptosis, and angiogenesis. J Immunother 23: 519–527. 11001545 10.1097/00002371-200009000-00002

[31] Yang Z, Wang WE, Zhang Q (2013) CIAPIN1 siRNA inhibits proliferation, migration and promotes apoptosis of VSMCs by regulating Bcl-2 and Bax. Curr Neurovasc Res 10: 4–10. 23151078 10.2174/156720213804805909

[32] Renault TT, Manon S (2011) Bax: Addressed to kill. Biochimie 93: 1379–1391. 21641962 10.1016/j.biochi.2011.05.013

[33] Jablonski EM, Webb AN, McConnell NA, Riley MC, Hughes FM Jr (2004) Plasma membrane aquaporin activity can affect the rate of apoptosis but is inhibited after apoptotic volume decrease. Am J Physiol Cell Physiol 286: C975–985. 14644770 10.1152/ajpcell.00180.2003

[34] Ammirante M, De Laurenzi V, Graziano V, Turco MC, Rosati A (2010) BAG3 is required for IKKalpha nuclear translocation and emergence of castration resistant prostate cancer. Cell Death Dis 2: e139. 10.1038/cddis.2011.23 PMC3101818 21451574

[35] Rosati A, Graziano V, De Laurenzi V, Pascale M, Turco MC (2011) BAG3: a multifaceted protein that regulates major cell pathways. Cell Death Dis 2: e141. 21472004 10.1038/cddis.2011.24 PMC3122056

[36] Festa M, Del Valle L, Khalili K, Franco R, Scognamiglio G, et al (2011) BAG3 protein is overexpressed in human glioblastoma and is a potential target for therapy. Am J Pathol 178: 2504–2512. 21561597 10.1016/j.ajpath.2011.02.002 PMC3124067

[37] Benjamin JM, Nelson WJ (2008) Bench to bedside and back again: molecular mechanisms of alpha-catenin function and roles in tumorigenesis. Semin Cancer Biol 18: 53–64. 17945508 10.1016/j.semcancer.2007.08.003 PMC2692220

[38] Cheung CT, Singh R, Yoon AR, Hasan MK, Yaguchi T, et al (2011) Molecular characterization of apoptosis induced by CARF silencing in human cancer cells. Cell Death Differ 18: 589–601. 21052095 10.1038/cdd.2010.129 PMC3131902

[39] Kaul SC, Hasan K, Wadhwa R (2006) CARF regulates p19ARF-p53-p21WAF1 senescence pathway by multiple checkpoints. Ann N Y Acad Sci 1067: 217–219. 16803988 10.1196/annals.1354.026

[40] Cheung CT, Hasan MK, Widodo N, Kaul SC, Wadhwa R (2009) CARF: an emerging regulator of p53 tumor suppressor and senescence pathway. Mech Ageing Dev 130: 18–23. 18555516 10.1016/j.mad.2008.05.002

[41] Marcel V, Dichtel-Danjoy ML, Sagne C, Hafsi H, Ma D, et al (2011) Biological functions of p53 isoforms through evolution: lessons from animal and cellular models. Cell Death Differ 18: 1815–1824. 21941372 10.1038/cdd.2011.120 PMC3214904

[42] Pietsch EC, Sykes SM, McMahon SB, Murphy ME (2008) The p53 family and programmed cell death. Oncogene 27: 6507–6521. 18955976 10.1038/onc.2008.315 PMC2657599

[43] McPherson JP, Goldenberg GJ (1998) Induction of apoptosis by deregulated expression of DNA topoisomerase IIalpha. Cancer Res 58: 4519–4524. 9788593

[44] Otera H, Ishihara N, Mihara K (2013) New insights into the function and regulation of mitochondrial fission. Biochim Biophys Acta 1833: 1256–1268. 23434681 10.1016/j.bbamcr.2013.02.002

[45] Hsu DK, Yang RY, Liu FT (2006) Galectins in apoptosis. Methods Enzymol 417: 256–273. 17132510 10.1016/S0076-6879(06)17018-4

[46] Joly AL, Wettstein G, Mignot G, Ghiringhelli F, Garrido C (2010) Dual role of heat shock proteins as regulators of apoptosis and innate immunity. J Innate Immun 2: 238–247. 20375559 10.1159/000296508

[47] Meares GP, Zmijewska AA, Jope RS (2008) HSP105 interacts with GRP78 and GSK3 and promotes ER stress-induced caspase-3 activation. Cell Signal 20: 347–358. 18083346 10.1016/j.cellsig.2007.10.032 PMC2212615

[48] Li Y, Shin D, Kwon SH (2013) Histone deacetylase 6 plays a role as a distinct regulator of diverse cellular processes. FEBS J 280: 775–793. 23181831 10.1111/febs.12079

[49] Sahlgren CM, Pallari HM, He T, Chou YH, Goldman RD, et al (2006) A nestin scaffold links Cdk5/p35 signaling to oxidant-induced cell death. EMBO J 25: 4808–4819. 17036052 10.1038/sj.emboj.7601366 PMC1618100

[50] Li Z, Hann SR (2009) The Myc-nucleophosmin-ARF network: a complex web unveiled. Cell Cycle 8: 2703–2707. 19652540 10.4161/cc.8.17.9418 PMC3234343

[51] Chae HZ, Oubrahim H, Park JW, Rhee SG, Chock PB (2012) Protein glutathionylation in the regulation of peroxiredoxins: a family of thiol-specific peroxidases that function as antioxidants, molecular chaperones, and signal modulators. Antioxid Redox Signal 16: 506–523. 22114845 10.1089/ars.2011.4260 PMC3270059

[52] Brigelius-Flohe R, Maiorino M (2013) Glutathione peroxidases. Biochim Biophys Acta 1830: 3289–3303. 23201771 10.1016/j.bbagen.2012.11.020

[53] Shahzidi S, Brech A, Sioud M, Li X, Suo Z, et al (2013) Lamin A/C cleavage by caspase-6 activation is crucial for apoptotic induction by photodynamic therapy with hexaminolevulinate in human B-cell lymphoma cells. Cancer Lett 339: 25–32. 23916608 10.1016/j.canlet.2013.07.026

[54] Fuller-Pace FV (2013) The DEAD box proteins DDX5 (p68) and DDX17 (p72): multi-tasking transcriptional regulators. Biochim Biophys Acta 1829: 756–763. 23523990 10.1016/j.bbagrm.2013.03.004

[55] Strappazzon F, Torch S, Chatellard-Causse C, Petiot A, Thibert C, et al (2010) Alix is involved in caspase 9 activation during calcium-induced apoptosis. Biochem Biophys Res Commun 397: 64–69. 20471954 10.1016/j.bbrc.2010.05.062

[56] Mahul-Mellier AL, Strappazzon F, Petiot A, Chatellard-Causse C, Torch S, et al (2008) Alix and ALG-2 are involved in tumor necrosis factor receptor 1-induced cell death. J Biol Chem 283: 34954–34965. 18936101 10.1074/jbc.M803140200 PMC3259881

[57] Wan Q, Kuang E, Dong W, Zhou S, Xu H, et al (2007) Reticulon 3 mediates Bcl-2 accumulation in mitochondria in response to endoplasmic reticulum stress. Apoptosis 12: 319–328. 17191123 10.1007/s10495-006-0574-y

[58] Inohara N, del Peso L, Koseki T, Chen S, Nunez G (1998) RICK, a novel protein kinase containing a caspase recruitment domain, interacts with CLARP and regulates CD95-mediated apoptosis. J Biol Chem 273: 12296–12300. 9575181 10.1074/jbc.273.20.12296

[59] Romeo Y, Zhang X, Roux PP (2012) Regulation and function of the RSK family of protein kinases. Biochem J 441: 553–569. 22187936 10.1042/BJ20110289

[60] Zhou A, Ou AC, Cho A, Benz EJ Jr, Huang SC (2008) Novel splicing factor RBM25 modulates Bcl-x pre-mRNA 5′ splice site selection. Mol Cell Biol 28: 5924–5936. 18663000 10.1128/MCB.00560-08 PMC2546994

[61] Seshacharyulu P, Pandey P, Datta K, Batra SK (2013) Phosphatase: PP2A structural importance, regulation and its aberrant expression in cancer. Cancer Lett 335: 9–18. 23454242 10.1016/j.canlet.2013.02.036 PMC3665613

[62] Yan L, Lavin VA, Moser LR, Cui Q, Kanies C, et al (2008) PP2A regulates the pro-apoptotic activity of FOXO1. J Biol Chem 283: 7411–7420. 18211894 10.1074/jbc.M708083200 PMC2276329

[63] Chen W, Wang H, Tao S, Zheng Y, Wu W, et al (2013) Tumor protein translationally controlled 1 is a p53 target gene that promotes cell survival. Cell Cycle 12: 2321–2328. 24067374 10.4161/cc.25404 PMC3755082

[64] Emami S (2011) Interplay between p53-family, their regulators, and PARPs in DNA repair. Clin Res Hepatol Gastroenterol 35: 98–104. 21177056 10.1016/j.gcb.2010.10.002

