# Supplementary material for: Identification of O-GlcNAc Modification Targets in Mouse Retinal Pericytes: Implication of p53 in Pathogenesis of Diabetic Retinopathy
Source: PLoS One. 2014 May 1;9(5):e95561. doi: 10.1371/journal.pone.0095561 (PMC4006792; doi:10.1371/journal.pone.0095561)
Supplement: File S1 — Proteins selectively identified in GlcNAlk samples by mass spectrometry. Data was considered high confidence (Table S1) if the number of assigned spectra was at least 10-fold greater for GlcNAlk samples compared with DMSO control samples. Further, the protein must have been identified with spectral counts greater than or equal to 5. Data was considered medium confidence (Table S2) if the number of assigned spectra was at least 2 fold greater for GlcNAlk samples than DMSO control samples. Further, the protein must have been identified with spectral counts greater than or equal to 2. Bolded proteins were not identified previously as O-GlcNAc modification target. (DOCX) [file pone.0095561.s001.docx]

**Supporting Information**

**Table S1**

| **Identified Proteins (190, 40 new)** | **Accession Number** | **GlcNAlk** | **DMSO** | **Citation** | **Functional Group** |
| --- | --- | --- | --- | --- | --- |
| DNA replication licensing factor MCM3 | IPI00108338 | 18 | 0 | [[1](#_ENREF_1),[2](#_ENREF_2)] | DNA replication |
| DNA replication licensing factor MCM4 | IPI00117016 | 13 | 0 | [[3](#_ENREF_3)] | DNA replication |
| Galectin-1 | IPI00229517 | 7 | 0 | [[1](#_ENREF_1),[2](#_ENREF_2)] | Extracellular Signaling |
| Myb-binding protein 1A | IPI00331361 | 25 | 5 | [[3](#_ENREF_3)] | Gene Regulation & Transcription |
| Matrin-3 | IPI00453826 | 15 | 0 | [[1](#_ENREF_1),[2](#_ENREF_2),[3](#_ENREF_3)] | Gene Regulation & Transcription |
| Histone H1.2 | IPI00223713 | 12 | 1 | [[3](#_ENREF_3)] | Gene Regulation & Transcription |
| Isoform Alpha of Lamina-associated polypeptide 2, isoforms alpha/zeta | IPI00126338 | 12 | 0 | [[1](#_ENREF_1),[2](#_ENREF_2),[3](#_ENREF_3)] | Gene Regulation & Transcription |
| Isoform 1 of Non-POU domain-containing octamer-binding protein | IPI00320016 | 8 | 0 | [[1](#_ENREF_1),[2](#_ENREF_2),[3](#_ENREF_3),[4](#_ENREF_4),[5](#_ENREF_5)] | Gene Regulation & Transcription |
| Host cell factor C1 | IPI00828490 | 6 | 0 | [[1](#_ENREF_1),[3](#_ENREF_3),[4](#_ENREF_4),[5](#_ENREF_5),[6](#_ENREF_6),[7](#_ENREF_7),[8](#_ENREF_8),[9](#_ENREF_9),[10](#_ENREF_10),[11](#_ENREF_11)] | Gene Regulation & Transcription |
| DNA ligase 1 | IPI00473314 | 5 | 0 | [[3](#_ENREF_3)] | Gene Regulation & Transcription |
| Histone H1.4 | IPI00223714 | 5 | 0 | [[3](#_ENREF_3),[4](#_ENREF_4)] | Gene Regulation & Transcription |
| Isoform 1 of Leucine-rich repeat flightless-interacting protein 1 | IPI00654388 | 6 | 0 | [[1](#_ENREF_1),[2](#_ENREF_2),[3](#_ENREF_3)] | Gene Regulation & Transcription |
| Cold shock domain-containing protein E1 isoform 2 | IPI00938510 | 12 | 0 | [[1](#_ENREF_1),[2](#_ENREF_2)] | Gene Regulation & Transcription |
| Signal transducer and activator of transcription 1 isoform 2 | IPI01016187 | 12 | 0 | [[1](#_ENREF_1),[2](#_ENREF_2)] | Gene Regulation & Transcription |
| Cellular tumor antigen p53 isoform b | IPI00648350 | 8 | 0 | [[12](#_ENREF_12)] | Gene Regulation & Transcription |
| **Histone H1.1** | **IPI00228616** | **7** | **0** |  | **Gene Regulation & Transcription** |
| **Structural maintenance of chromosomes protein** | **IPI00857414** | **6** | **0** |  | **Gene Regulation & Transcription** |
| TAR DNA-binding protein 43 | IPI00989821 | 6 | 0 | [[3](#_ENREF_3),[13](#_ENREF_13)] | Gene Regulation & Transcription |
| Transcriptional activator protein Pur-alpha | IPI00118447 | 6 | 0 | [[1](#_ENREF_1),[2](#_ENREF_2),[14](#_ENREF_14)] | Gene Regulation & Transcription |
| **Interferon-inducible GTPase 1** | **IPI00323496** | **27** | **5** |  | **Immunity** |
| SAM domain and HD domain-containing protein 1 | IPI00120095 | 16 | 0 | [[1](#_ENREF_1),[2](#_ENREF_2)] | Immunity |
| **Isoform 2 of Immunity-related GTPase family M protein 1** | **IPI00889294** | **11** | **2** |  | **Immunity** |
| **Isoform 1 of Z-DNA-binding protein 1** | **IPI00135469** | **9** | **0** |  | **Immunity** |
| **H-2 class I histocompatibility antigen, D-B alpha chain** | **IPI01026878** | **8** | **0** |  | **Immunity** |
| **Interferon-induced guanylate-binding protein 2** | **IPI00323251** | **8** | **1** |  | **Immunity** |
| **H-2 class I histocompatibility antigen, K-W28 alpha chain** | **IPI00974711** | **6** | **0** |  | **Immunity** |
| **Interferon gamma inducible protein 47** | **IPI00123570** | **6** | **0** |  | **Immunity** |
| Receptor-interacting serine/threonine-protein kinase 2 | IPI00139382 | 6 | 0 | [[1](#_ENREF_1),[2](#_ENREF_2)] | Immunity |
| ATP-binding cassette sub-family E member 1 | IPI00322869 | 13 | 0 | [[1](#_ENREF_1),[2](#_ENREF_2),[3](#_ENREF_3)] | Intracellular Trafficking |
| Voltage-dependent anion-selective channel protein 3 | IPI00122548 | 10 | 0 | [[1](#_ENREF_1),[2](#_ENREF_2),[14](#_ENREF_14)] | Intracellular Trafficking |
| Vesicle-associated membrane protein-associated protein A | IPI00125267 | 7 | 0 | [[3](#_ENREF_3)] | Intracellular Trafficking |
| **Isoform SERCA2A of Sarcoplasmic/endoplasmic reticulum calcium ATPase 2** | **IPI00468900** | **6** | **0** |  | **Intracellular Trafficking** |
| Nuclear mitotic apparatus protein 1 | IPI00263048 | 6 | 0 | [[1](#_ENREF_1),[2](#_ENREF_2),[13](#_ENREF_13)] | Intracellular Trafficking |
| **Sodium/potassium-transporting ATPase subunit alpha-1** | **IPI00311682** | **6** | **0** |  | **Intracellular Trafficking** |
| Isoform 2 of T-complex protein 1 subunit alpha | IPI00845611 | 5 | 0 | [[1](#_ENREF_1),[2](#_ENREF_2)] | Intracellular Trafficking |
| Thioredoxin-like protein 1 | IPI00266281 | 5 | 0 | [[1](#_ENREF_1),[2](#_ENREF_2)] | Intracellular Trafficking |
| Isoform M2 of Pyruvate kinase isozymes M1/M2 | IPI00407130 | 23 | 4 | [[1](#_ENREF_1),[3](#_ENREF_3),[4](#_ENREF_4),[11](#_ENREF_11),[13](#_ENREF_13),[15](#_ENREF_15),[16](#_ENREF_16),[17](#_ENREF_17),[18](#_ENREF_18)] | Metabolism |
| L-lactate dehydrogenase A chain | IPI00319994 | 17 | 0 | [[1](#_ENREF_1),[2](#_ENREF_2),[3](#_ENREF_3),[5](#_ENREF_5),[16](#_ENREF_16)] | Metabolism |
| Asparagine synthetase [glutamine-hydrolyzing] | IPI00116966 | 16 | 0 | [[1](#_ENREF_1),[2](#_ENREF_2),[3](#_ENREF_3)] | Metabolism |
| Glyceraldehyde-3-phosphate dehydrogenase | IPI00989722 | 13 | 5 | [[1](#_ENREF_1),[2](#_ENREF_2),[3](#_ENREF_3),[4](#_ENREF_4),[7](#_ENREF_7),[16](#_ENREF_16),[17](#_ENREF_17),[19](#_ENREF_19)] | Metabolism |
| GMP synthase [glutamine-hydrolyzing] | IPI00351252 | 13 | 0 | [[1](#_ENREF_1),[2](#_ENREF_2),[3](#_ENREF_3)] | Metabolism |
| Inosine-5'-monophosphate dehydrogenase 2 | IPI00323971 | 12 | 0 | [[1](#_ENREF_1),[2](#_ENREF_2),[3](#_ENREF_3),[4](#_ENREF_4),[13](#_ENREF_13)] | Metabolism |
| ADP/ATP translocase 2 | IPI00127841 | 11 | 3 | [[2](#_ENREF_2),[3](#_ENREF_3),[4](#_ENREF_4),[15](#_ENREF_15)] | Metabolism |
| Multifunctional protein ADE2 | IPI00322096 | 11 | 0 | [[1](#_ENREF_1),[2](#_ENREF_2),[3](#_ENREF_3),[4](#_ENREF_4)] | Metabolism |
| Trifunctional purine biosynthetic protein adenosine-3 | IPI00323644 | 10 | 0 | [[1](#_ENREF_1),[2](#_ENREF_2),[3](#_ENREF_3)] | Metabolism |
| Inorganic pyrophosphatase | IPI00110684 | 9 | 0 | [[1](#_ENREF_1),[2](#_ENREF_2),[3](#_ENREF_3)] | Metabolism |
| Triosephosphate isomerase | IPI00467833 | 9 | 0 | [[1](#_ENREF_1),[2](#_ENREF_2),[3](#_ENREF_3),[4](#_ENREF_4),[15](#_ENREF_15),[18](#_ENREF_18),[19](#_ENREF_19)] | Metabolism |
| Isoform 2 of Thioredoxin reductase 1, cytoplasmic | IPI00988125 | 8 | 0 | [[1](#_ENREF_1),[2](#_ENREF_2),[3](#_ENREF_3)] | Metabolism |
| Ribonucleoside-diphosphate reductase large subunit | IPI00315127 | 8 | 0 | [[1](#_ENREF_1),[2](#_ENREF_2),[3](#_ENREF_3),[13](#_ENREF_13)] | Metabolism |
| S-methyl-5'-thioadenosine phosphorylase | IPI00132096 | 8 | 0 | [[1](#_ENREF_1),[2](#_ENREF_2),[3](#_ENREF_3),[13](#_ENREF_13)] | Metabolism |
| D-3-phosphoglycerate dehydrogenase | IPI00225961 | 7 | 0 | [[1](#_ENREF_1),[2](#_ENREF_2),[3](#_ENREF_3),[13](#_ENREF_13),[15](#_ENREF_15)] | Metabolism |
| Cytochrome b5 type B | IPI00315794 | 5 | 0 | [[3](#_ENREF_3)] | Metabolism |
| Peroxiredoxin-1 | IPI00121788 | 5 | 0 | [[1](#_ENREF_1),[2](#_ENREF_2),[3](#_ENREF_3)] | Metabolism |
| Peroxiredoxin-4 | IPI00116254 | 5 | 0 | [[1](#_ENREF_1),[2](#_ENREF_2),[3](#_ENREF_3),[13](#_ENREF_13)] | Metabolism |
| Uridine 5'-monophosphate synthase | IPI00121552 | 5 | 0 | [[3](#_ENREF_3)] | Metabolism |
| **Acyl-coenzyme A thioesterase 9, mitochondrial** | **IPI00314069** | **14** | **0** |  | **Metabolism** |
| **ADP/ATP translocase 1** | **IPI00115564** | **10** | **3** |  | **Metabolism** |
| Fatty acid synthase | IPI00113223 | 8 | 0 | [[1](#_ENREF_1),[2](#_ENREF_2)] | Metabolism |
| Isoform 1 of Inorganic pyrophosphatase 2, mitochondrial | IPI00127050 | 8 | 0 | [[1](#_ENREF_1),[2](#_ENREF_2)] | Metabolism |
| Hydroxymethylglutaryl-CoA synthase, cytoplasmic | IPI00331707 | 7 | 0 | [[1](#_ENREF_1),[2](#_ENREF_2)] | Metabolism |
| **Inositol monophosphatase 1** | **IPI00625248** | **7** | **0** |  | **Metabolism** |
| Isoform 1 of Opioid growth factor receptor | IPI00121018 | 6 | 0 | [[1](#_ENREF_1),[2](#_ENREF_2)] | Metabolism |
| **Lanosterol synthase** | **IPI00169958** | **6** | **0** |  | **Metabolism** |
| Reticulon-4 isoform B1 | IPI00470167 | 6 | 0 | [[6](#_ENREF_6),[14](#_ENREF_14)] | Metabolism |
| Cad protein | IPI00620956 | 5 | 0 | [[1](#_ENREF_1),[2](#_ENREF_2),[13](#_ENREF_13)] | Metabolism |
| Deoxyuridine triphosphatase isoform 2 | IPI00467266 | 5 | 0 | [[1](#_ENREF_1),[2](#_ENREF_2)] | Metabolism |
| Glycerol-3-phosphate dehydrogenase, mitochondrial | IPI00331182 | 5 | 0 | [[1](#_ENREF_1),[2](#_ENREF_2)] | Metabolism |
| GrpE protein homolog 1, mitochondrial | IPI00117083 | 5 | 0 | [[1](#_ENREF_1),[2](#_ENREF_2)] | Metabolism |
| Mitochondrial inner membrane protein | IPI00990313 | 5 | 0 | [[1](#_ENREF_1),[2](#_ENREF_2)] | Metabolism |
| Mitochondrial-processing peptidase subunit beta | IPI00274656 | 5 | 0 | [[1](#_ENREF_1),[2](#_ENREF_2)] | Metabolism |
| Elongation factor 2 | IPI00466069 | 28 | 0 | [[1](#_ENREF_1),[2](#_ENREF_2),[3](#_ENREF_3),[4](#_ENREF_4),[15](#_ENREF_15)] | Protein Synthesis, Quality Control, & Turnover |
| T-complex protein 1 subunit beta | IPI00320217 | 22 | 0 | [[1](#_ENREF_1),[2](#_ENREF_2),[3](#_ENREF_3),[15](#_ENREF_15)] | Protein Synthesis, Quality Control, & Turnover |
| Serpin B6 | IPI00121471 | 16 | 0 | [[3](#_ENREF_3)] | Protein Synthesis, Quality Control, & Turnover |
| Nucleolar protein 58 | IPI00463468 | 15 | 0 | [[1](#_ENREF_1),[2](#_ENREF_2),[3](#_ENREF_3)] | Protein Synthesis, Quality Control, & Turnover |
| E3 ubiquitin-protein ligase NEDD4 | IPI00462445 | 12 | 0 | [[3](#_ENREF_3),[6](#_ENREF_6),[14](#_ENREF_14)] | Protein Synthesis, Quality Control, & Turnover |
| Elongation factor 1-gamma | IPI00318841 | 9 | 0 | [[1](#_ENREF_1),[2](#_ENREF_2),[3](#_ENREF_3)] | Protein Synthesis, Quality Control, & Turnover |
| Heat shock protein HSP 90-alpha | IPI00330804 | 9 | 0 | [[1](#_ENREF_1),[2](#_ENREF_2),[3](#_ENREF_3),[4](#_ENREF_4),[13](#_ENREF_13)] | Protein Synthesis, Quality Control, & Turnover |
| Proteasome activator complex subunit 2 | IPI00124225 | 9 | 0 | [[1](#_ENREF_1),[2](#_ENREF_2),[3](#_ENREF_3),[13](#_ENREF_13)] | Protein Synthesis, Quality Control, & Turnover |
| SUMO-activating enzyme subunit 2 | IPI00130173 | 7 | 0 | [[1](#_ENREF_1),[2](#_ENREF_2),[3](#_ENREF_3)] | Protein Synthesis, Quality Control, & Turnover |
| 60S ribosomal protein L10a | IPI00127085 | 5 | 0 | [[3](#_ENREF_3),[4](#_ENREF_4)] | Protein Synthesis, Quality Control, & Turnover |
| Active regulator of SIRT1 | IPI00226227 | 5 | 0 | [[3](#_ENREF_3)] | Protein Synthesis, Quality Control, & Turnover |
| Glutathione S-transferase omega-1 | IPI00114285 | 5 | 0 | [[1](#_ENREF_1),[2](#_ENREF_2),[3](#_ENREF_3),[18](#_ENREF_18)] | Protein Synthesis, Quality Control, & Turnover |
| Bifunctional aminoacyl-tRNA synthetase | IPI00339916 | 24 | 2 | [[1](#_ENREF_1),[2](#_ENREF_2),[3](#_ENREF_3)] | Protein Synthesis, Quality Control, & Turnover |
| Glycyl-tRNA synthetase | IPI00112555 | 12 | 0 | [[1](#_ENREF_1),[2](#_ENREF_2),[3](#_ENREF_3),[13](#_ENREF_13)] | Protein Synthesis, Quality Control, & Turnover |
| Alanyl-tRNA synthetase, cytoplasmic | IPI00321308 | 8 | 0 | [[1](#_ENREF_1),[2](#_ENREF_2),[3](#_ENREF_3)] | Protein Synthesis, Quality Control, & Turnover |
| Ubiquitin carboxyl-terminal hydrolase | IPI00881918 | 16 | 0 | [[1](#_ENREF_1),[2](#_ENREF_2)] | Protein Synthesis, Quality Control, & Turnover |
| Prolyl endopeptidase | IPI00989777 | 14 | 0 | [[1](#_ENREF_1),[2](#_ENREF_2)] | Protein Synthesis, Quality Control, & Turnover |
| Isoform Short of Delta-1-pyrroline-5-carboxylate synthase | IPI00221398 | 13 | 0 | [[1](#_ENREF_1),[2](#_ENREF_2)] | Protein Synthesis, Quality Control, & Turnover |
| Ubiquitin-like modifier-activating enzyme 1 | IPI00123313 | 10 | 0 | [[1](#_ENREF_1),[2](#_ENREF_2),[13](#_ENREF_13)] | Protein Synthesis, Quality Control, & Turnover |
| Valyl-tRNA synthetase | IPI00130353 | 10 | 0 | [[1](#_ENREF_1),[2](#_ENREF_2),[13](#_ENREF_13)] | Protein Synthesis, Quality Control, & Turnover |
| **AH receptor-interacting protein** | **IPI00117178** | **9** | **0** |  | **Protein Synthesis, Quality Control, & Turnover** |
| Nuclear pore complex protein Nup155 | IPI00453821 | 8 | 0 | [[1](#_ENREF_1),[2](#_ENREF_2)] | Protein Synthesis, Quality Control, & Turnover |
| **Ribosomal L1 domain containing 1** | **IPI00989654** | **8** | **0** |  | **Protein Synthesis, Quality Control, & Turnover** |
| Ribosome-binding protein 1 isoform a | IPI00755120 | 8 | 0 | [[1](#_ENREF_1),[2](#_ENREF_2)] | Protein Synthesis, Quality Control, & Turnover |
| **40S ribosomal protein S11** | **IPI00762542** | **7** | **2** |  | **Protein Synthesis, Quality Control, & Turnover** |
| 40S ribosomal protein S9 | IPI00420726 | 7 | 2 | [[20](#_ENREF_20)] | Protein Synthesis, Quality Control, & Turnover |
| Isoform C of Fragile X mental retardation syndrome-related protein 1 | IPI00230599 | 7 | 0 | [[1](#_ENREF_1),[2](#_ENREF_2)] | Protein Synthesis, Quality Control, & Turnover |
| Protein phosphatase 1G | IPI00117072 | 7 | 0 | [[1](#_ENREF_1),[2](#_ENREF_2)] | Protein Synthesis, Quality Control, & Turnover |
| **Ras-related protein Rab-7a** | **IPI00408892** | **7** | **0** |  | **Protein Synthesis, Quality Control, & Turnover** |
| Calpain-2 catalytic subunit | IPI00308938 | 6 | 0 | [[1](#_ENREF_1),[2](#_ENREF_2)] | Protein Synthesis, Quality Control, & Turnover |
| **Cullin-3** | **IPI00467383** | **6** | **0** |  | **Protein Synthesis, Quality Control, & Turnover** |
| Isoform 1 of Ubiquitin carboxyl-terminal hydrolase 10 | IPI00955687 | 6 | 0 | [[1](#_ENREF_1),[2](#_ENREF_2)] | Protein Synthesis, Quality Control, & Turnover |
| **Proteasome subunit beta type-8** | **IPI00116712** | **6** | **0** |  | **Protein Synthesis, Quality Control, & Turnover** |
| **Ras-related protein Rab-14** | **IPI00126042** | **6** | **0** |  | **Protein Synthesis, Quality Control, & Turnover** |
| **60S ribosomal protein L36** | **IPI00463297** | **5** | **0** |  | **Protein Synthesis, Quality Control, & Turnover** |
| Isoform 2 of Tryptophanyl-tRNA synthetase, cytoplasmic | IPI00355382 | 5 | 0 | [[1](#_ENREF_1),[2](#_ENREF_2)] | Protein Synthesis, Quality Control, & Turnover |
| Mitochondrial import receptor subunit TOM40 homolog | IPI00474157 | 5 | 0 | [[1](#_ENREF_1),[2](#_ENREF_2)] | Protein Synthesis, Quality Control, & Turnover |
| **Thimet oligopeptidase** | **IPI00322150** | **5** | **0** |  | **Protein Synthesis, Quality Control, & Turnover** |
| Ubiquitin-conjugating enzyme E2 L3-like | IPI00116718 | 5 | 0 | [[1](#_ENREF_1),[2](#_ENREF_2)] | Protein Synthesis, Quality Control, & Turnover |
| Poly(rC)-binding protein 1 | IPI00128904 | 9 | 0 | [[1](#_ENREF_1),[2](#_ENREF_2),[3](#_ENREF_3),[13](#_ENREF_13)] | RNA Processing |
| Far upstream element-binding protein 2 | IPI00462934 | 22 | 0 | [[1](#_ENREF_1),[2](#_ENREF_2),[3](#_ENREF_3),[13](#_ENREF_13)] | RNA Processing |
| Nucleolar RNA helicase 2 | IPI00120691 | 22 | 0 | [[1](#_ENREF_1),[2](#_ENREF_2),[3](#_ENREF_3)] | RNA Processing |
| Putative pre-mRNA-splicing factor ATP-dependent RNA Processing helicase DHX15 | IPI00128818 | 22 | 0 | [[1](#_ENREF_1),[2](#_ENREF_2),[3](#_ENREF_3)] | RNA Processing |
| Heterogeneous nuclear ribonucleoprotein F | IPI00798511 | 10 | 0 | [[1](#_ENREF_1),[2](#_ENREF_2),[3](#_ENREF_3)] | RNA Processing |
| KH domain-containing, RNA-binding, signal transduction-associated protein 1 | IPI00458765 | 7 | 0 | [[1](#_ENREF_1),[2](#_ENREF_2),[3](#_ENREF_3)] | RNA Processing |
| ATP-dependent RNA helicase DDX24 isoform 1 | IPI00380228 | 5 | 0 | [[3](#_ENREF_3)] | RNA Processing |
| Isoform 1 of Probable ATP-dependent RNA helicase DDX17 | IPI00396797 | 5 | 0 | [[1](#_ENREF_1),[2](#_ENREF_2),[3](#_ENREF_3)] | RNA Processing |
| Polypyrimidine tract binding protein 1 | IPI00136883 | 10 | 0 | [[1](#_ENREF_1),[2](#_ENREF_2),[3](#_ENREF_3)] | RNA Processing |
| ELAV-like protein 1 | IPI00108271 | 5 | 0 | [[1](#_ENREF_1),[2](#_ENREF_2),[3](#_ENREF_3),[4](#_ENREF_4)] | RNA Processing |
| **Processing of precursor 1 isoform 2** | **IPI00172251** | **15** | **0** |  | **RNA Processing** |
| Heterogeneous nuclear ribonucleoprotein K | IPI00990152 | 12 | 0 | [[1](#_ENREF_1),[2](#_ENREF_2),[13](#_ENREF_13)] | RNA Processing |
| Isoform 2 of Heterogeneous nuclear ribonucleoprotein M | IPI00480357 | 8 | 0 | [[1](#_ENREF_1),[2](#_ENREF_2)] | RNA Processing |
| Isoform 2 of RNA-binding protein 25 | IPI00928541 | 6 | 0 | [[1](#_ENREF_1),[2](#_ENREF_2)] | RNA Processing |
| **Poly(A)-specific ribonuclease PARN** | **IPI00311534** | **6** | **0** |  | **RNA Processing** |
| **Poly(rC)-binding protein 2 isoform 4** | **IPI00956778** | **6** | **0** |  | **RNA Processing** |
| **Putative ATP-dependent RNA helicase Pl10** | **IPI00133708** | **6** | **0** |  | **RNA Processing** |
| Ribosome biogenesis protein BOP1 | IPI00653297 | 6 | 0 | [[1](#_ENREF_1),[2](#_ENREF_2)] | RNA Processing |
| Heterogeneous nuclear ribonucleoprotein H | IPI00133916 | 5 | 0 | [[1](#_ENREF_1),[2](#_ENREF_2)] | RNA Processing |
| Heterogeneous nuclear ribonucleoprotein H2 | IPI00108143 | 5 | 0 | [[1](#_ENREF_1),[2](#_ENREF_2)] | RNA Processing |
| Pre-mRNA branch site protein p14 | IPI00117687 | 5 | 0 | [[1](#_ENREF_1),[2](#_ENREF_2)] | RNA Processing |
| AHNAK nucleoprotein isoform 1 | IPI00553798 | 41 | 6 | [[1](#_ENREF_1),[2](#_ENREF_2),[3](#_ENREF_3)] | Signaling |
| Annexin A1 | IPI00230395 | 12 | 0 | [[3](#_ENREF_3),[4](#_ENREF_4),[13](#_ENREF_13)] | Signaling |
| 14-3-3 protein epsilon | IPI00118384 | 11 | 0 | [[1](#_ENREF_1),[2](#_ENREF_2),[3](#_ENREF_3),[15](#_ENREF_15),[18](#_ENREF_18)] | Signaling |
| Isoform 1 of 14-3-3 protein theta | IPI00656269 | 9 | 0 | [[1](#_ENREF_1),[2](#_ENREF_2),[15](#_ENREF_15),[17](#_ENREF_17)] | Signaling |
| Calponin-2 | IPI00116649 | 8 | 0 | [[1](#_ENREF_1),[2](#_ENREF_2),[3](#_ENREF_3)] | Signaling |
| 14-3-3 protein zeta/delta | IPI00116498 | 5 | 0 | [[1](#_ENREF_1),[2](#_ENREF_2),[3](#_ENREF_3),[17](#_ENREF_17)] | Signaling |
| Isoform 2 of Adenylate kinase 2, mitochondrial | IPI00269076 | 5 | 0 | [[1](#_ENREF_1),[2](#_ENREF_2),[3](#_ENREF_3),[13](#_ENREF_13)] | Signaling |
| **ADP-ribosylation factor 1** | **IPI00221613** | **8** | **0** |  | **Signaling** |
| Nucleophosmin | IPI00127415 | 8 | 0 | [[1](#_ENREF_1),[2](#_ENREF_2),[13](#_ENREF_13)] | Signaling |
| Heat shock protein HSP 90-beta | IPI00554929 | 19 | 6 | [[1](#_ENREF_1),[2](#_ENREF_2),[13](#_ENREF_13)] | Stress response |
| **Isoform Cytoplasmic of Phospholipid hydroperoxide glutathione peroxidase, mitochondrial** | **IPI00989123** | **7** | **0** |  | **Stress response** |
| DnaJ homolog subfamily C member 7 | IPI00331385 | 6 | 0 | [[1](#_ENREF_1),[2](#_ENREF_2)] | Stress response |
| Isoform 2 of Heat shock 70 kDa protein 4L | IPI00317711 | 5 | 0 | [[1](#_ENREF_1),[2](#_ENREF_2)] | Stress response |
| Isoform 1 of Filamin-A | IPI00875567 | 85 | 17 | [[1](#_ENREF_1),[2](#_ENREF_2),[3](#_ENREF_3)] | Structural |
| Myosin-9 | IPI00123181 | 61 | 12 | [[1](#_ENREF_1),[2](#_ENREF_2),[3](#_ENREF_3),[4](#_ENREF_4),[13](#_ENREF_13),[19](#_ENREF_19)] | Structural |
| Isoform A of Lamin-A/C | IPI00620256 | 38 | 4 | [[1](#_ENREF_1),[2](#_ENREF_2),[3](#_ENREF_3),[6](#_ENREF_6)] | Structural |
| Filamin-B | IPI00663627 | 35 | 0 | [[1](#_ENREF_1),[2](#_ENREF_2),[3](#_ENREF_3)] | Structural |
| Isoform 2 of Tropomyosin alpha-3 chain | IPI00230044 | 21 | 3 | [[3](#_ENREF_3)] | Structural |
| Annexin A2 | IPI00468203 | 20 | 5 | [[1](#_ENREF_1),[2](#_ENREF_2),[3](#_ENREF_3),[15](#_ENREF_15),[17](#_ENREF_17)] | Structural |
| Adenylyl cyclase-associated protein 1 | IPI0013733 | 12 | 0 | [[1](#_ENREF_1),[2](#_ENREF_2),[3](#_ENREF_3)] | Structural |
| caldesmon 1 | IPI00122450 | 12 | 0 | [[1](#_ENREF_1),[2](#_ENREF_2),[3](#_ENREF_3)] | Structural |
| LIM domain and actin-binding protein 1 isoform a | IPI00112339 | 12 | 0 | [[1](#_ENREF_1),[2](#_ENREF_2),[3](#_ENREF_3)] | Structural |
| microtubule-associated protein 4 | IPI00848819 | 11 | 0 | [[1](#_ENREF_1),[2](#_ENREF_2),[3](#_ENREF_3),[4](#_ENREF_4),[6](#_ENREF_6),[13](#_ENREF_13),[14](#_ENREF_14),[15](#_ENREF_15)] | Structural |
| Tubulin beta-5 chain | IPI00117352 | 10 | 3 | [[3](#_ENREF_3),[4](#_ENREF_4),[15](#_ENREF_15),[17](#_ENREF_17),[18](#_ENREF_18)] | Structural |
| Isoform Smooth muscle of Myosin light polypeptide 6 | IPI00354819 | 7 | 0 | [[1](#_ENREF_1),[2](#_ENREF_2),[3](#_ENREF_3),[13](#_ENREF_13)] | Structural |
| Actin-binding protein anillin | IPI00172197 | 6 | 0 | [[1](#_ENREF_1),[2](#_ENREF_2),[3](#_ENREF_3)] | Structural |
| Destrin | IPI00127942 | 5 | 0 | [[1](#_ENREF_1),[2](#_ENREF_2),[3](#_ENREF_3)] | Structural |
| Alpha-actinin-1a | IPI00989903 | 40 | 0 | [[1](#_ENREF_1),[2](#_ENREF_2)] | Structural |
| Filamin-C | IPI00753917 | 35 | 0 | [[4](#_ENREF_4)] | Structural |
| Alpha-actinin-4 | IPI00118899 | 27 | 0 | [[1](#_ENREF_1),[2](#_ENREF_2)] | Structural |
| Ankycorbin | IPI00453820 | 14 | 0 | [[1](#_ENREF_1),[2](#_ENREF_2)] | Structural |
| Isoform 1 of Nestin | IPI00453692 | 13 | 0 | [[1](#_ENREF_1),[2](#_ENREF_2)] | Structural |
| Tubulin alpha-1A chain | IPI00110753 | 12 | 3 | [[21](#_ENREF_21)] | Structural |
| Tropomyosin alpha-4 chain | IPI00421223 | 10 | 2 | [[1](#_ENREF_1),[2](#_ENREF_2),[3](#_ENREF_3)] | Structural |
| Isoform 3 of Palladin | IPI00856926 | 9 | 0 | [[1](#_ENREF_1),[2](#_ENREF_2)] | Structural |
| **Capping protein (Actin filament) muscle Z-line, beta** | **IPI00776140** | **7** | **0** |  | **Structural** |
| Cofilin-1-like | IPI00848816 | 7 | 0 | [[1](#_ENREF_1),[2](#_ENREF_2)] | Structural |
| **Cytoskeleton-associated protein 4** | **IPI00223047** | **6** | **0** |  | **Structural** |
| Inverted formin-2 | IPI01007967 | 6 | 0 | [[1](#_ENREF_1),[2](#_ENREF_2)] | Structural |
| Isoform E2 of Drebrin | IPI00331516 | 5 | 0 | [[1](#_ENREF_1),[2](#_ENREF_2)] | Structural |
| Src substrate cortactin | IPI00118143 | 5 | 0 | [[1](#_ENREF_1),[2](#_ENREF_2),[3](#_ENREF_3),[4](#_ENREF_4)] | Structural |
| T-complex protein 1 subunit theta | IPI00469268 | 5 | 0 | [[1](#_ENREF_1),[2](#_ENREF_2),[13](#_ENREF_13)] | Structural |
| Tropomodulin-3 | IPI00119478 | 5 | 0 | [[1](#_ENREF_1),[2](#_ENREF_2)] | Structural |
| 60 kDa protein | IPI00985815 | 11 | 0 | [[1](#_ENREF_1),[2](#_ENREF_2)] | Unknown |
| Prmt1 | IPI00974841 | 11 | 0 | [[1](#_ENREF_1),[2](#_ENREF_2),[13](#_ENREF_13)] | Unknown |
| 30 kDa protein | IPI01026712 | 10 | 0 | [[1](#_ENREF_1),[2](#_ENREF_2)] | Unknown |
| **22 kDa protein** | **IPI00989100** | **9** | **0** |  | **Unknown** |
| **Uncharacterized protein** | **IPI00466185** | **8** | **2** |  | **Unknown** |
| Eif4b | IPI00985742 | 7 | 0 | [[1](#_ENREF_1),[2](#_ENREF_2)] | Unknown |
| **NAD-dependent malic enzyme, mitochondrial-like** | **IPI00987622** | **7** | **0** |  | **Unknown** |
| 16 kDa protein | IPI00648714 | 6 | 0 | [[1](#_ENREF_1),[2](#_ENREF_2)] | Unknown |
| **23 kDa protein** | **IPI00775915** | **6** | **3** |  | **Unknown** |
| Heterogenous nuclear ribonucleoprotein U | IPI00970121 | 6 | 0 | [[1](#_ENREF_1),[2](#_ENREF_2)] | Unknown |
| High mobility group protein B1-like | IPI00665601 | 6 | 0 | [[1](#_ENREF_1),[2](#_ENREF_2),[3](#_ENREF_3)] | Unknown |
| **55 kDa erythrocyte membrane protein** | **IPI00856759** | **5** | **0** |  | **Unknown** |
| Ataxin-2-like protein | IPI00988540 | 5 | 0 | [[1](#_ENREF_1),[2](#_ENREF_2)] | Unknown |
| HEAT repeat-containing protein 3 | IPI00399686 | 5 | 0 | [[1](#_ENREF_1),[2](#_ENREF_2)] | Unknown |
| **Leucine-rich repeat-containing protein 59** | **IPI00653744** | **5** | **0** |  | **Unknown** |
| **Uncharacterized protein** | **IPI00115992** | **5** | **2** |  | **Unknown** |
| **Uncharacterized protein C19 or f43 homolog** | **IPI00133958** | **5** | **0** |  | **Unknown** |

**Table S2.**

| **Identified Proteins (241, 75 new)** | **Accession Number** | **GlcNAlk** | **DMSO** | **Citation** | **Functional Group** |
| --- | --- | --- | --- | --- | --- |
| Protein RCC2 | IPI00222509 | 4 | 0 | [[1](#_ENREF_1),[2](#_ENREF_2)] | DNA replication |
| **DNA topoisomerase 2-alpha** | **IPI00122223** | **3** | **0** |  | **DNA replication** |
| Mini-chromosome maintenance complex-binding protein | IPI00153724 | 2 | 0 | [[1](#_ENREF_1),[2](#_ENREF_2)] | DNA replication |
| DNA mismatch repair protein Msh2 | IPI00118158 | 2 | 0 | [[1](#_ENREF_1),[2](#_ENREF_2)] | DNA replication |
| Isoform 3 of Septin-11 | IPI00420385 | 3 | 0 | [[1](#_ENREF_1),[2](#_ENREF_2)] | Gene Regulation & Transcription |
| Protein flightless-1 homolog | IPI00467104 | 2 | 0 | [[1](#_ENREF_1),[2](#_ENREF_2)] | Gene Regulation & Transcription |
| BAG family molecular chaperone regulator 3 | IPI00331334 | 2 | 0 | [[1](#_ENREF_1),[2](#_ENREF_2),[3](#_ENREF_3)] | Gene Regulation & Transcription |
| Translationally-controlled tumor protein | IPI00918599 | 2 | 0 | [[3](#_ENREF_3)] | Gene Regulation & Transcription |
| Isoform 2 of KN motif and ankyrin repeat domain-containing protein 2 | IPI00761961 | 2 | 0 | [[6](#_ENREF_6)] | Gene Regulation & Transcription |
| Anamorsin | IPI00187301 | 3 | 0 | [[1](#_ENREF_1),[2](#_ENREF_2),[3](#_ENREF_3)] | Gene Regulation & Transcription |
| Hepatoma-derived growth factor | IPI00313817 | 4 | 0 | [[1](#_ENREF_1),[2](#_ENREF_2),[3](#_ENREF_3)] | Gene Regulation & Transcription |
| Nucleolin | IPI00317794 | 3 | 0 | [[1](#_ENREF_1),[2](#_ENREF_2),[3](#_ENREF_3),[4](#_ENREF_4)] | Gene Regulation & Transcription |
| ATP-dependent RNA helicase DDX1 | IPI00127172 | 2 | 0 | [[1](#_ENREF_1),[2](#_ENREF_2)] | Gene Regulation & Transcription |
| Thioredoxin | IPI00226993 | 2 | 0 | [[1](#_ENREF_1),[2](#_ENREF_2),[3](#_ENREF_3),[4](#_ENREF_4)] | Gene Regulation & Transcription |
| Isoform 2 of PRKC apoptosis WT1 regulator protein | IPI00816941 | 4 | 0 | [[1](#_ENREF_1),[2](#_ENREF_2)] | Gene Regulation & Transcription |
| Isoform C of Serrate RNA effector molecule homolog | IPI00224644 | 2 | 0 | [[1](#_ENREF_1),[2](#_ENREF_2)] | Gene Regulation & Transcription |
| CDKN2A-interacting protein | IPI00263028 | 2 | 0 | [[1](#_ENREF_1),[2](#_ENREF_2)] | Gene Regulation & Transcription |
| Isoform 1 of Poly [ADP-ribose] polymerase 1 | IPI00112473 | 2 | 0 | [[1](#_ENREF_1),[2](#_ENREF_2),[13](#_ENREF_13)] | Gene Regulation & Transcription |
| Structural maintenance of chromosomes flexible hinge domain-containing protein 1 | IPI00137433 | 2 | 0 | [[1](#_ENREF_1),[2](#_ENREF_2)] | Gene Regulation & Transcription |
| Chromobox protein homolog 3, HP1γ | IPI00129468 | 4 | 0 | [[1](#_ENREF_1),[2](#_ENREF_2),[3](#_ENREF_3)] | Gene Regulation & Transcription |
| **Protein Zfp326** | **IPI00986915** | **4** | **0** |  | **Gene Regulation & Transcription** |
| Chromatin assembly factor 1 subunit A | IPI00133839 | 3 | 0 | [[1](#_ENREF_1),[2](#_ENREF_2)] | Gene Regulation & Transcription |
| High mobility group protein B2 | IPI00462291 | 3 | 0 | [[1](#_ENREF_1),[2](#_ENREF_2),[3](#_ENREF_3)] | Gene Regulation & Transcription |
| High mobility group protein HMGI-C | IPI00331612 | 3 | 0 | [[3](#_ENREF_3)] | Gene Regulation & Transcription |
| **Interferon-activable protein 205-A** | **IPI00319489** | **3** | **0** |  | **Gene Regulation & Transcription** |
| Chromobox protein homolog 5 | IPI00123755 | 2 | 0 | [[1](#_ENREF_1),[2](#_ENREF_2),[3](#_ENREF_3)] | Gene Regulation & Transcription |
| Chromodomain-helicase-DNA-binding protein 4 | IPI00857777 | 2 | 0 | [[1](#_ENREF_1),[2](#_ENREF_2)] | Gene Regulation & Transcription |
| Ribosomal RNA-processing protein 8 | IPI00119166 | 2 | 0 | [[1](#_ENREF_1),[2](#_ENREF_2),[14](#_ENREF_14)] | Gene Regulation & Transcription |
| Ewing sarcoma breakpoint region 1 | IPI00515199 | 2 | 0 | [[3](#_ENREF_3),[13](#_ENREF_13),[22](#_ENREF_22)] | Gene Regulation & Transcription |
| **Von Willebrand factor A domain-containing protein 5A** | **IPI00221817** | **3** | **0** |  | **Gene Regulation & Transcription** |
| Histone deacetylase 6 | IPI00775830 | 2 | 0 | [[1](#_ENREF_1),[2](#_ENREF_2)] | Gene Regulation & Transcription |
| **Septin 9** | **IPI00648786** | **2** | **0** |  | **Gene Regulation & Transcription** |
| **Bone marrow stromal antigen 2** | **IPI00321222** | **3** | **0** |  | **Immunity** |
| **Interferon-induced transmembrane protein 3** | **IPI00133243** | **3** | **0** |  | **Immunity** |
| Stomatin-like protein 2 | IPI00115117 | 4 | 0 | [[1](#_ENREF_1),[2](#_ENREF_2)] | Immunity |
| T-complex protein 1 subunit epsilon | IPI00116279 | 2 | 0 | [[1](#_ENREF_1),[2](#_ENREF_2)] | Immunity |
| T-complex protein 1 subunit zeta | IPI00116281 | 2 | 0 | [[1](#_ENREF_1),[2](#_ENREF_2),[13](#_ENREF_13)] | Immunity |
| aminoacyl tRNA synthase complex-interacting multifunctional protein 1 | IPI00132194 | 3 | 0 | [[6](#_ENREF_6)] | Immunity |
| **Isoform 1 of Probable ATP-dependent RNA helicase DDX58** | **IPI00273914** | **2** | **0** |  | **Immunity** |
| **Tapasin** | **IPI00226205** | **3** | **0** |  | **Immunity** |
| **Isoform 1 of Transmembrane emp24 domain-containing protein 10** | **IPI00466570** | **4** | **1** |  | **Intracellular Trafficking** |
| **Mitotic spindle assembly checkpoint protein MAD2A** | **IPI00323422** | **3** | **0** |  | **Intracellular Trafficking** |
| **Isoform 4 of Dynamin-1-like protein** | **IPI00556857** | **4** | **0** |  | **Intracellular Trafficking** |
| Isoform 1 of Ras-related GTP-binding protein C | IPI00468702 | 3 | 0 | [[1](#_ENREF_1),[2](#_ENREF_2)] | Intracellular Trafficking |
| **Ras-related protein Rab-2A** | **IPI00137227** | **4** | **0** |  | **Intracellular Trafficking** |
| **Ras-related protein Rab-10** | **IPI00130118** | **4** | **0** |  | **Intracellular Trafficking** |
| **Receptor expression-enhancing protein 5** | **IPI00315463** | **4** | **0** |  | **Intracellular Trafficking** |
| **Phosphate carrier protein, mitochondrial** | **IPI00124771** | **4** | **0** |  | **Intracellular Trafficking** |
| Nuclear pore complex protein Nup214 | IPI00988948 | 2 | 0 | [[1](#_ENREF_1)] | Intracellular Trafficking |
| Nucleoporin 153 | IPI00330624 | 2 | 0 | [[2](#_ENREF_2)] | Intracellular Trafficking |
| **Nucleolar protein 16** | **IPI00458704** | **4** | **1** |  | **Intracellular Trafficking** |
| SEC23-interacting protein | IPI00648142 | 3 | 0 | [[3](#_ENREF_3),[4](#_ENREF_4),[13](#_ENREF_13)] | Intracellular Trafficking |
| **Nucleoside diphosphate kinase B** | **IPI00127417** | **3** | **0** |  | **Metabolism** |
| Protein disulfide-isomerase | IPI00133522 | 3 | 0 | [[1](#_ENREF_1),[2](#_ENREF_2)] | Metabolism |
| Glutamate--cysteine ligase regulatory subunit | IPI00114329 | 3 | 0 | [[1](#_ENREF_1),[2](#_ENREF_2),[3](#_ENREF_3)] | Metabolism |
| Phosphoribosylformylglycinamidine synthase | IPI00265406 | 3 | 0 | [[1](#_ENREF_1),[2](#_ENREF_2)] | Metabolism |
| Isoform 2 of A-kinase anchor protein 12 | IPI00867770 | 2 | 0 | [[1](#_ENREF_1),[2](#_ENREF_2)] | Metabolism |
| **N-acetylneuraminic acid synthase (Sialic acid synthase)** | **IPI00114925** | **2** | **0** |  | **Metabolism** |
| Phosphoserine phosphatase | IPI00117146 | 2 | 0 | [[3](#_ENREF_3)] | Metabolism |
| Branched-chain-amino-acid aminotransferase | IPI00875896 | 2 | 0 | [[1](#_ENREF_1),[2](#_ENREF_2),[3](#_ENREF_3)] | Metabolism |
| Hexokinase-2 | IPI00989451 | 2 | 0 | [[1](#_ENREF_1),[2](#_ENREF_2)] | Metabolism |
| CTP synthase 1 | IPI00111959 | 4 | 0 | [[1](#_ENREF_1),[2](#_ENREF_2),[3](#_ENREF_3)] | Metabolism |
| Aldose reductase-related protein 2 | IPI00273096 | 3 | 0 | [[3](#_ENREF_3)] | Metabolism |
| Isoform A of Cytosolic acyl coenzyme A thioester hydrolase | IPI00284094 | 3 | 0 | [[1](#_ENREF_1),[2](#_ENREF_2),[14](#_ENREF_14)] | Metabolism |
| Aldose reductase | IPI00223757 | 2 | 0 | [[1](#_ENREF_1),[2](#_ENREF_2)] | Metabolism |
| ATP-citrate synthase | IPI00762047 | 2 | 0 | [[1](#_ENREF_1),[2](#_ENREF_2),[13](#_ENREF_13)] | Metabolism |
| Choline-phosphate cytidylyltransferase A | IPI00857041 | 2 | 0 | [[1](#_ENREF_1),[2](#_ENREF_2)] | Metabolism |
| **Isoform 1 of Estradiol 17-beta-dehydrogenase 12** | **IPI00119219** | **2** | **0** |  | **Metabolism** |
| Malate dehydrogenase, mitochondrial | IPI00323592 | 2 | 0 | [[2](#_ENREF_2),[13](#_ENREF_13)] | Metabolism |
| **Prostaglandin E synthase 2** | **IPI00312174** | **3** | **0** |  | **Metabolism** |
| Prostaglandin E synthase 3 | IPI00985716 | 3 | 0 | [[1](#_ENREF_1),[2](#_ENREF_2)] | Metabolism |
| Isoform Cytoplasmic of Glutathione reductase, mitochondrial | IPI00760002 | 4 | 1 | [[1](#_ENREF_1),[2](#_ENREF_2)] | Metabolism |
| **5'-AMP-activated protein kinase catalytic subunit alpha-1** | **IPI00556823** | **2** | **0** |  | **Metabolism** |
| Glucose-6-phosphate isomerase | IPI00228633 | 3 | 0 | [[1](#_ENREF_1),[2](#_ENREF_2),[13](#_ENREF_13)] | Metabolism |
| Cytochrome c-type heme lyase | IPI00134572 | 2 | 0 | [[1](#_ENREF_1),[2](#_ENREF_2)] | Metabolism |
| Dihydrolipoyllysine-residue acetyltransferase component of pyruvate dehydrogenase complex, mitochondrial | IPI00153660 | 2 | 0 | [[1](#_ENREF_1),[2](#_ENREF_2)] | Metabolism |
| isopentenyl-diphosphate Delta-isomerase 1 | IPI00987185 | 3 | 0 | [[3](#_ENREF_3)] | Metabolism |
| **Catenin alpha-1** | **IPI00112963** | **3** | **0** |  | **Metabolism** |
| Protein disulfide-isomerase A3 | IPI00230108 | 3 | 0 | [[1](#_ENREF_1),[2](#_ENREF_2)] | Metabolism |
| **Trans-2,3-enoyl-CoA reductase** | **IPI00262743** | **3** | **0** |  | **Metabolism** |
| Zyx protein | IPI00408218 | 4 | 0 | [[1](#_ENREF_1),[2](#_ENREF_2),[6](#_ENREF_6)] | Metabolism |
| Membrane-associated progesterone receptor component 1 | IPI00986206 | 2 | 0 | [[1](#_ENREF_1),[2](#_ENREF_2)] | Metabolism |
| Mitochondrial carrier homolog 2 | IPI00132039 | 3 | 0 | [[1](#_ENREF_1),[2](#_ENREF_2)] | Metabolism |
| **MOSC domain-containing protein 2, mitochondrial** | **IPI00123276** | **2** | **0** |  | **Metabolism** |
| **Trimethyllysine dioxygenase, mitochondrial** | **IPI00129163** | **4** | **0** |  | **Metabolism** |
| Isoform 3 of Glyoxalase domain-containing protein 4 | IPI00112630 | 3 | 0 | [[1](#_ENREF_1),[2](#_ENREF_2)] | Metabolism |
| **Pyruvate carboxylase, mitochondrial isoform 2** | **IPI00114710** | **3** | **0** |  | **Metabolism** |
| Protein Mki67 | IPI00124959 | 2 | 0 | [[1](#_ENREF_1),[2](#_ENREF_2)] | Metabolism |
| Splicing factor 1 isoform 2 | IPI00830797 | 2 | 0 | [[1](#_ENREF_1),[2](#_ENREF_2)] | Metabolism |
| **Large neutral amino acids transporter small subunit 1** | **IPI00129395** | **2** | **0** |  | **Protein Synthesis, Quality Control, & Turnover** |
| Isoform 1 of Protein phosphatase 1 regulatory subunit 12A | IPI00876048 | 2 | 0 | [[1](#_ENREF_1),[2](#_ENREF_2),[4](#_ENREF_4),[14](#_ENREF_14),[15](#_ENREF_15)] | Protein Synthesis, Quality Control, & Turnover |
| Protein phosphatase 1 regulatory subunit 11 | IPI00851027 | 2 | 0 | [[3](#_ENREF_3)] | Protein Synthesis, Quality Control, & Turnover |
| STIP1 homology and U box-containing protein 1 | IPI00471361 | 2 | 0 | [[1](#_ENREF_1),[2](#_ENREF_2)] | Protein Synthesis, Quality Control, & Turnover |
| ***NAD+ ADP-ribosyltransferase 3 PARP-3*** | ***IPI00874306*** | ***2*** | ***0*** |  | ***Protein Synthesis, Quality Control, & Turnover*** |
| Cystatin-B | IPI00125931 | 2 | 0 | [[1](#_ENREF_1),[2](#_ENREF_2)] | Protein Synthesis, Quality Control, & Turnover |
| **DET1- and DDB1-associated protein 1** | **IPI00896596** | **2** | **0** |  | **Protein Synthesis, Quality Control, & Turnover** |
| Ubiquitin thioesterase OTUB1 | IPI00928020 | 3 | 0 | [[1](#_ENREF_1),[2](#_ENREF_2),[3](#_ENREF_3)] | Protein Synthesis, Quality Control, & Turnover |
| Eukaryotic peptide chain release factor GTP-binding subunit ERF3A isoform 2 | IPI00911165 | 2 | 0 | [[1](#_ENREF_1),[2](#_ENREF_2)] | Protein Synthesis, Quality Control, & Turnover |
| Eukaryotic translation initiation factor 2 subunit 3, X-linked | IPI00775852 | 2 | 0 | [[3](#_ENREF_3)] | Protein Synthesis, Quality Control, & Turnover |
| Eukaryotic translation initiation factor 6 | IPI00115862 | 2 | 0 | [[1](#_ENREF_1),[2](#_ENREF_2)] | Protein Synthesis, Quality Control, & Turnover |
| Arginyl-tRNA synthetase, cytoplasmic | IPI00315488 | 4 | 0 | [[1](#_ENREF_1),[2](#_ENREF_2)] | Protein Synthesis, Quality Control, & Turnover |
| Proteasome assembly chaperone 1 | IPI00885524 | 4 | 0 | [[1](#_ENREF_1),[2](#_ENREF_2)] | Protein Synthesis, Quality Control, & Turnover |
| 60S ribosomal protein L12 | IPI00849793 | 3 | 0 | [[1](#_ENREF_1),[2](#_ENREF_2)] | Protein Synthesis, Quality Control, & Turnover |
| 60S ribosomal protein L36a | IPI00225066 | 3 | 0 | [[4](#_ENREF_4)] | Protein Synthesis, Quality Control, & Turnover |
| 40S ribosomal protein S12 | IPI00225634 | 3 | 0 | [[1](#_ENREF_1),[2](#_ENREF_2),[3](#_ENREF_3)] | Protein Synthesis, Quality Control, & Turnover |
| **40S ribosomal protein S17** | **IPI00465880** | **3** | **0** |  | **Protein Synthesis, Quality Control, & Turnover** |
| 40S ribosomal protein S19 | IPI00113241 | 3 | 0 | [[1](#_ENREF_1),[2](#_ENREF_2),[3](#_ENREF_3)] | Protein Synthesis, Quality Control, & Turnover |
| **Cytosolic non-specific dipeptidase** | **IPI00315879** | **3** | **0** |  | **Protein Synthesis, Quality Control, & Turnover** |
| Eukaryotic initiation factor 4A-I isoform 2 | IPI00929797 | 3 | 0 | [[1](#_ENREF_1),[2](#_ENREF_2)] | Protein Synthesis, Quality Control, & Turnover |
| Glycylpeptide N-tetradecanoyltransferase 1 | IPI00224128 | 3 | 0 | [[1](#_ENREF_1),[2](#_ENREF_2)] | Protein Synthesis, Quality Control, & Turnover |
| Leucyl-tRNA synthetase, cytoplasmic | IPI00453819 | 3 | 0 | [[1](#_ENREF_1),[2](#_ENREF_2),[3](#_ENREF_3)] | Protein Synthesis, Quality Control, & Turnover |
| SEC24 related gene family, member D | IPI00284595 | 2 | 0 | [[1](#_ENREF_1),[2](#_ENREF_2)] | Protein Synthesis, Quality Control, & Turnover |
| **Mitochondrial import inner membrane translocase subunit TIM50** | **IPI00111045** | **2** | **0** |  | **Protein Synthesis, Quality Control, & Turnover** |
| **Vesicle-trafficking protein SEC22b** | **IPI00880617** | **2** | **0** |  | **Protein Synthesis, Quality Control, & Turnover** |
| Isoform 1 of Programmed cell death 6-interacting protein | IPI00453818 | 3 | 0 | [[1](#_ENREF_1),[2](#_ENREF_2)] | Protein Synthesis, Quality Control, & Turnover |
| 40S ribosomal protein S3a | IPI00331345 | 2 | 0 | [[1](#_ENREF_1),[2](#_ENREF_2)] | Protein Synthesis, Quality Control, & Turnover |
| 40S ribosomal protein S5 | IPI00125521 | 2 | 0 | [[1](#_ENREF_1),[2](#_ENREF_2)] | Protein Synthesis, Quality Control, & Turnover |
| **60S ribosomal protein L23-like** | **IPI00849782** | **2** | **0** |  | **Protein Synthesis, Quality Control, & Turnover** |
| **60S ribosomal protein L30-like** | **IPI00463886** | **2** | **0** |  | **Protein Synthesis, Quality Control, & Turnover** |
| 60S ribosomal protein L37 | IPI00453924 | 2 | 0 | [[4](#_ENREF_4)] | Protein Synthesis, Quality Control, & Turnover |
| Eukaryotic translation initiation factor 3 subunit G | IPI00622371 | 4 | 0 | [[1](#_ENREF_1),[2](#_ENREF_2),[3](#_ENREF_3),[4](#_ENREF_4)] | Protein Synthesis, Quality Control, & Turnover |
| N-alpha-acetyltransferase 15, NatA auxiliary subunit | IPI00387212 | 4 | 1 | [[1](#_ENREF_1),[2](#_ENREF_2),[3](#_ENREF_3)] | Protein Synthesis, Quality Control, & Turnover |
| **Polyubiquitin-B** | **IPI00139518** | **4** | **0** |  | **Protein Synthesis, Quality Control, & Turnover** |
| 60S acidic ribosomal protein P0 | IPI00314950 | 3 | 0 | [[1](#_ENREF_1),[2](#_ENREF_2),[3](#_ENREF_3),[4](#_ENREF_4)] | Protein Synthesis, Quality Control, & Turnover |
| **Ubiquitin-like protein ISG15** | **IPI00555085** | **3** | **0** |  | **Protein Synthesis, Quality Control, & Turnover** |
| Isoform 3 of Presequence protease, mitochondrial, Pitrilysin metalloproteinase 1 | IPI00788353 | 4 | 0 | [[1](#_ENREF_1),[18](#_ENREF_18)] | Protein Synthesis, Quality Control, & Turnover |
| Calnexin | IPI00119618 | 4 | 0 | [[1](#_ENREF_1),[2](#_ENREF_2),[3](#_ENREF_3)] | Protein Synthesis, Quality Control, & Turnover |
| Serine/threonine-protein phosphatase 4 regulatory subunit 2 | IPI00756355 | 2 | 0 | [[1](#_ENREF_1),[2](#_ENREF_2)] | Protein Synthesis, Quality Control, & Turnover |
| Isoform 3 of Reticulon-3 | IPI00112948 | 2 | 0 | [[6](#_ENREF_6),[14](#_ENREF_14)] | Protein Synthesis, Quality Control, & Turnover |
| **Ras-related protein Rab-18** | **IPI00116770** | **2** | **0** |  | **Protein Synthesis, Quality Control, & Turnover** |
| **Isoform 1 of 39S ribosomal protein L17, mitochondrial** | **IPI00111784** | **3** | **0** |  | **Protein Synthesis, Quality Control, & Turnover** |
| Serine/threonine-protein phosphatase 2A 65 kDa regulatory subunit A alpha isoform | IPI00310091 | 4 | 0 | [[1](#_ENREF_1),[2](#_ENREF_2),[13](#_ENREF_13)] | Protein Synthesis, Quality Control, & Turnover |
| Nuclear factor 90 | IPI00776384 | 2 | 0 | [[1](#_ENREF_1),[2](#_ENREF_2)] | Protein Synthesis, Quality Control, & Turnover |
| E3 SUMO-protein ligase RanBP2 | IPI00337844 | 2 | 0 | [[1](#_ENREF_1),[2](#_ENREF_2),[14](#_ENREF_14)] | Protein Synthesis, Quality Control, & Turnover |
| 60S ribosomal protein L11 | IPI00331461 | 3 | 0 | [[3](#_ENREF_3)] | Protein Synthesis, Quality Control, & Turnover |
| Sec24 related gene family, member C isoform 2 | IPI00626620 | 2 | 0 | [[4](#_ENREF_4)] | Protein Synthesis, Quality Control, & Turnover |
| Treacle protein | IPI00408190 | 3 | 0 | [[1](#_ENREF_1),[2](#_ENREF_2)] | Protein Synthesis, Quality Control, & Turnover |
| **Isoform 1 of Cyclin-dependent kinase inhibitor 2A, isoforms 1/2** | **IPI00283736** | **2** | **0** |  | **Protein Synthesis, Quality Control, & Turnover** |
| UBX domain-containing protein 4 | IPI00165799 | 2 | 0 | [[1](#_ENREF_1),[2](#_ENREF_2)] | Protein Synthesis, Quality Control, & Turnover |
| Calcyclin-binding protein | IPI00115650 | 4 | 0 | [[3](#_ENREF_3)] | Protein Synthesis, Quality Control, & Turnover |
| **26S proteasome non-ATPase regulatory subunit 6** | **IPI00319965** | **2** | **0** |  | **Protein Synthesis, Quality Control, & Turnover** |
| 26S proteasome non-ATPase regulatory subunit 2 | IPI00123494 | 3 | 0 | [[1](#_ENREF_1),[2](#_ENREF_2),[4](#_ENREF_4)] | Protein Synthesis, Quality Control, & Turnover |
| **Putative uncharacterized protein** | **IPI00380130** | **4** | **0** |  | **Protein Synthesis, Quality Control, & Turnover** |
| Caprin-1 isoform c | IPI00121515 | 4 | 1 | [[1](#_ENREF_1),[2](#_ENREF_2),[3](#_ENREF_3),[4](#_ENREF_4)] | Protein Synthesis, Quality Control, & Turnover |
| Isoform 1 of COP9 signalosome complex subunit 7a | IPI00123465 | 3 | 0 | [[1](#_ENREF_1),[2](#_ENREF_2),[3](#_ENREF_3)] | Protein Sythesis, Processing & Trafficking |
| Eukaryotic translation initiation factor 3 subunit E | IPI00132250 | 3 | 0 | [[1](#_ENREF_1),[2](#_ENREF_2)] | Protein Sythesis, Processing & Trafficking |
| Eukaryotic translation initiation factor 4 gamma 1 | IPI00856453 | 3 | 0 | [[1](#_ENREF_1),[2](#_ENREF_2),[13](#_ENREF_13)] | Protein Sythesis, Processing & Trafficking |
| **Isoform 2 of E3 ubiquitin-protein ligase DTX3L** | **IPI00856986** | **3** | **0** |  | **Protein Sythesis, Processing & Trafficking** |
| Isoform Short of Tripeptidyl-peptidase 2 | IPI00227843 | 3 | 0 | [[1](#_ENREF_1),[2](#_ENREF_2)] | Protein Sythesis, Processing & Trafficking |
| Ran-binding protein 3 | IPI00135190 | 3 | 0 | [[1](#_ENREF_1),[2](#_ENREF_2)] | Protein Sythesis, Processing & Trafficking |
| Nuclear pore complex protein Nup50 | IPI00120572 | 3 | 0 | [[1](#_ENREF_1),[2](#_ENREF_2),[4](#_ENREF_4)] | Protein Sythesis, Processing & Trafficking |
| Guanine nucleotide-binding protein G(i) subunit alpha-2 | IPI00228617 | 3 | 0 | [[3](#_ENREF_3)] | RNA Processing |
| Ribonuclease inhibitor | IPI00313296 | 2 | 0 | [[1](#_ENREF_1),[2](#_ENREF_2)] | RNA Processing |
| Ribosomal protein S6 kinase alpha-3 | IPI00831226 | 4 | 0 | [[1](#_ENREF_1),[2](#_ENREF_2)] | RNA Processing |
| Isoform 1 of La-related protein 1 | IPI00929786 | 4 | 1 | [[1](#_ENREF_1),[2](#_ENREF_2),[3](#_ENREF_3)] | RNA Processing |
| Isoform 1 of tRNA (cytosine(34)-C(5))-methyltransferase | IPI00894870 | 3 | 0 | [[1](#_ENREF_1),[2](#_ENREF_2)] | RNA Processing |
| Isoform 3 of Plasminogen activator inhibitor 1 RNA-binding protein, PAI1 RNA-binding protein 1, PAI-RBP1 SERPINE1 mRNA-binding protein 1 | IPI00471477 | 4 | 0 | [[1](#_ENREF_1),[2](#_ENREF_2)] | RNA Processing |
| Isoform 4 of Double-stranded RNA-specific adenosine deaminase | IPI00762162 | 2 | 0 | [[1](#_ENREF_1),[2](#_ENREF_2)] | RNA Processing |
| Isoform Long of Heterogeneous nuclear ribonucleoprotein A1 | IPI00817004 | 2 | 0 | [[1](#_ENREF_1),[2](#_ENREF_2)] | RNA Processing |
| 40S ribosomal protein S15a | IPI00857457 | 4 | 0 | [[1](#_ENREF_1),[2](#_ENREF_2)] | RNA Processing |
| **40S ribosomal protein S13** | **IPI00989181** | **4** | **0** |  | **RNA Processing** |
| 60S ribosomal protein L17 | IPI00453768 | 4 | 0 | [[4](#_ENREF_4)] | RNA Processing |
| Nup98 protein | IPI00474558 | 3 | 0 | [[1](#_ENREF_1),[2](#_ENREF_2),[3](#_ENREF_3),[14](#_ENREF_14)] | RNA Processing |
| **Isoform 4 of Serine/arginine-rich splicing factor 7** | **IPI00474169** | **2** | **0** |  | **RNA Processing** |
| **Small nuclear ribonucleoprotein Sm D1** | **IPI00322749** | **2** | **0** |  | **RNA Processing** |
| **Protein RRP5 homolog** | **IPI00551454** | **2** | **0** |  | **RNA Processing** |
| Transducin beta-like protein 3 | IPI00124057 | 2 | 0 | [[1](#_ENREF_1),[2](#_ENREF_2)] | RNA Processing |
| Isoform 2 of Pre-mRNA-processing factor 40 homolog A | IPI00338887 | 4 | 0 | [[1](#_ENREF_1),[2](#_ENREF_2)] | RNA Processing |
| Enhancer of mRNA-decapping protein 4 | IPI00894741 | 3 | 0 | [[1](#_ENREF_1),[2](#_ENREF_2)] | RNA Processing |
| Isoform 2 of 5'-3' exoribonuclease 2 | IPI00283057 | 3 | 0 | [[1](#_ENREF_1),[2](#_ENREF_2)] | RNA Processing |
| Lupus La protein homolog | IPI00134300 | 3 | 0 | [[1](#_ENREF_1),[2](#_ENREF_2)] | RNA Processing |
| **60S ribosome subunit biogenesis protein NIP7 homolog isoform 2** | **IPI00944801** | **2** | **0** |  | **RNA Processing** |
| FUS interacting protein (Serine-arginine rich) 1 | IPI00649867 | 2 | 0 | [[1](#_ENREF_1),[2](#_ENREF_2)] | RNA Processing |
| **Isoform 2 of 40S ribosomal protein S24** | **IPI00402981** | **2** | **0** |  | **RNA Processing** |
| **Ribonuclease P protein subunit p30** | **IPI00134346** | **2** | **0** |  | **RNA Processing** |
| **Ribosome production factor 2 homolog isoform 2** | **IPI00776077** | **2** | **0** |  | **RNA Processing** |
| **Isoform 1 of RNA-binding protein Raly** | **IPI00308402** | **4** | **1** |  | **RNA Processing** |
| **Splicing factor 3A subunit 3** | **IPI00137848** | **3** | **0** |  | **RNA Processing** |
| **60S ribosomal protein L35a** | **IPI00115902** | **4** | **0** |  | **RNA Processing** |
| Isoform 1 of La-related protein 7 | IPI00340860 | 3 | 0 | [[1](#_ENREF_1),[2](#_ENREF_2)] | RNA Processing |
| **Isoform 2 of Myoferlin** | **IPI00677130** | **2** | **0** |  | **Signaling** |
| Ras-related protein Rab-5C | IPI00224518 | 4 | 0 | [[1](#_ENREF_1),[2](#_ENREF_2),[3](#_ENREF_3)] | Signaling |
| **Ras-related protein R-Ras2** | **IPI00323822** | **3** | **0** |  | **Signaling** |
| TBC1 domain family member 15 | IPI00110247 | 2 | 0 | [[1](#_ENREF_1),[2](#_ENREF_2)] | Signaling |
| **Transmembrane protein 109** | **IPI00919199** | **2** | **0** |  | **Signaling** |
| Signal recognition particle 19, isoform CRA_c | IPI00885711 | 2 | 0 | [[1](#_ENREF_1),[2](#_ENREF_2)] | Signaling |
| **Signal recognition particle receptor subunit beta** | **IPI00111271** | **2** | **0** |  | **Signaling** |
| Ran-specific GTPase-activating protein (Fragment) | IPI00321978 | 3 | 0 | [[1](#_ENREF_1),[2](#_ENREF_2),[3](#_ENREF_3),[4](#_ENREF_4)] | Signaling |
| **Tyrosine-protein phosphatase non-receptor type 2** | **IPI00885300** | **2** | **0** |  | **Signaling** |
| Dynactin subunit 2 | IPI00116112 | 3 | 0 | [[1](#_ENREF_1),[2](#_ENREF_2)] | Signaling |
| Apoptosis regulator BAX | IPI00990275 | 2 | 0 | [[3](#_ENREF_3)] | Signaling |
| Peflin | IPI00307963 | 3 | 0 | [[1](#_ENREF_1),[2](#_ENREF_2)] | Signaling |
| Isoform 2 of Rab GDP dissociation inhibitor beta | IPI00416577 | 2 | 0 | [[1](#_ENREF_1),[2](#_ENREF_2),[13](#_ENREF_13)] | Signaling |
| Aquaporin-1 | IPI00123183 | 3 | 0 | [[23](#_ENREF_23)] | Signaling |
| Ras GTPase-activating-like protein IQGAP1 | IPI00467447 | 2 | 0 | [[1](#_ENREF_1),[2](#_ENREF_2),[13](#_ENREF_13)] | Signaling |
| **Cytochrome c oxidase subunit 2** | **IPI00131176** | **3** | **0** |  | **Signaling** |
| Peroxiredoxin-2 | IPI00117910 | 3 | 0 | [[1](#_ENREF_1),[2](#_ENREF_2)] | Stress response |
| Heat shock 70 kDa protein 4 | IPI00331556 | 3 | 0 | [[1](#_ENREF_1),[2](#_ENREF_2),[13](#_ENREF_13)] | Stress response |
| Serpin H1 | IPI00114733 | 2 | 0 | [[1](#_ENREF_1),[2](#_ENREF_2)] | Stress response |
| Isoform HSP105-beta of Heat shock protein 105 kDa | IPI00224109 | 3 | 0 | [[1](#_ENREF_1),[2](#_ENREF_2),[4](#_ENREF_4)] | Stress response |
| Isoform 4 of Lipoma-preferred partner homolog | IPI00656290 | 3 | 0 | [[1](#_ENREF_1),[2](#_ENREF_2),[14](#_ENREF_14)] | Structural |
| Coatomer subunit beta | IPI00989020 | 2 | 0 | [[1](#_ENREF_1),[2](#_ENREF_2),[13](#_ENREF_13)] | Structural |
| Src substrate cortactin | IPI00118143 | 4 | 2 | [[1](#_ENREF_1),[2](#_ENREF_2),[3](#_ENREF_3),[4](#_ENREF_4)] | Structural |
| Dihydropyrimidinase-related protein 2 | IPI00114375 | 3 | 0 | [[8](#_ENREF_8),[14](#_ENREF_14)] | Structural |
| Spartin | IPI00880402 | 3 | 0 | [[6](#_ENREF_6)] | Structural |
| Calponin-3 | IPI00119111 | 2 | 0 | [[1](#_ENREF_1),[2](#_ENREF_2)] | Structural |
| Kinesin light chain 1 | IPI00623075 | 2 | 0 | [[1](#_ENREF_1),[2](#_ENREF_2)] | Structural |
| Tubulin-specific chaperone E | IPI00229931 | 2 | 0 | [[1](#_ENREF_1),[2](#_ENREF_2)] | Structural |
| Microtubule-associated protein 1S | IPI00223621 | 4 | 1 | [[1](#_ENREF_1),[2](#_ENREF_2),[3](#_ENREF_3)] | Structural |
| Cytosolic Fe-S cluster assembly factor NUBP2 | IPI00126925 | 3 | 0 | [[1](#_ENREF_1),[2](#_ENREF_2)] | Structural |
| Coronin-1C | IPI00124820 | 3 | 0 | [[1](#_ENREF_1),[2](#_ENREF_2),[3](#_ENREF_3),[13](#_ENREF_13)] | Structural |
| Lamin-B receptor | IPI00331173 | 3 | 0 | [[22](#_ENREF_22)] | Structural |
| Cysteine and glycine-rich protein 1 | IPI00123891 | 2 | 0 | [[1](#_ENREF_1),[2](#_ENREF_2),[13](#_ENREF_13)] | Structural |
| **SH3 domain-binding glutamic acid-rich-like protein** | **IPI00122265** | **4** | **0** |  | **Unknown** |
| Isoform 3 of OCIA domain-containing protein 1 | IPI00856842 | 2 | 0 | [[1](#_ENREF_1),[2](#_ENREF_2)] | Unknown |
| **Neighbor of COX4** | **IPI00117416** | **2** | **0** |  | **Unknown** |
| Protein FAM98B | IPI00465946 | 2 | 0 | [[1](#_ENREF_1),[2](#_ENREF_2),[3](#_ENREF_3),[4](#_ENREF_4)] | Unknown |
| RIKEN cDNA 1700009N14 gene | IPI00127109 | 2 | 0 | [[1](#_ENREF_1),[2](#_ENREF_2),[3](#_ENREF_3)] | Unknown |
| **Interferon-activable protein 204** | **IPI01008307** | **4** | **0** |  | **Unknown** |
| **La-related protein 4 isoform 2** | **IPI00828610** | **4** | **1** |  | **Unknown** |
| Nucleolar protein 56 | IPI00970722 | 4 | 2 | [[3](#_ENREF_3)] | Unknown |
| **50 kDa protein** | **IPI01027389** | **3** | **0** |  | **Unknown** |
| **OTU domain-containing protein 4** | **IPI01027056** | **3** | **0** |  | **Unknown** |
| Peptidyl-prolyl cis-trans isomerase | IPI00987580 | 3 | 0 | [[1](#_ENREF_1),[2](#_ENREF_2)] | Unknown |
| **Putative uncharacterized protein** | **IPI00990594** | **3** | **0** |  | **Unknown** |
| Putative uncharacterized protein (Fragment) | IPI00135308 | 3 | 0 | [[1](#_ENREF_1),[2](#_ENREF_2)] | Unknown |
| **12 kDa protein** | **IPI01027115** | **2** | **0** |  | **Unknown** |
| **17 kDa protein** | **IPI01026698** | **2** | **0** |  | **Unknown** |
| **26 kDa protein** | **IPI01026728** | **2** | **0** |  | **Unknown** |
| Gasdermin-D | IPI00111986 | 2 | 0 | [[1](#_ENREF_1),[2](#_ENREF_2)] | Unknown |
| Gem-associated protein 5 isoform 4 | IPI00649872 | 2 | 0 | [[1](#_ENREF_1),[2](#_ENREF_2)] | Unknown |
| Importin subunit alpha | IPI00985573 | 2 | 0 | [[3](#_ENREF_3),[13](#_ENREF_13)] | Unknown |
| **MKIAA0074 protein** | **IPI00875385** | **2** | **0** |  | **Unknown** |
| **Protein disulfide-isomerase A6-like** | **IPI00854971** | **2** | **0** |  | **Unknown** |
| Putative uncharacterized protein | IPI00474820 | 2 | 0 | [[1](#_ENREF_1),[2](#_ENREF_2),[14](#_ENREF_14)] | Unknown |
| **Putative uncharacterized protein** | **IPI00970004** | **2** | **0** |  | **Unknown** |
| **Tetratricopeptide repeat protein 1** | **IPI00130475** | **2** | **0** |  | **Unknown** |
| **Uncharacterized protein (Fragment)** | **IPI00988582** | **2** | **0** |  | **Unknown** |
| Uncharacterized protein (Fragment) | IPI00985637 | 2 | 0 | [[1](#_ENREF_1),[2](#_ENREF_2)] | Unknown |
| Uncharacterized protein (Fragment) | IPI00987441 | 2 | 0 |  | Unknown |
| UPF0552 protein C15orf38 homolog | IPI00461011 | 2 | 0 |  | Unknown |

**Supporting Information References**

1. Hahne H, Sobotzki N, Nyberg T, Helm D, Borodkin VS, et al. (2013) Proteome wide purification and identification of O-GlcNAc-modified proteins using click chemistry and mass spectrometry. J Proteome Res 12: 927-936.

2. Wang Z, Udeshi ND, Slawson C, Compton PD, Sakabe K, et al. (2010) Extensive crosstalk between O-GlcNAcylation and phosphorylation regulates cytokinesis. Sci Signal 3: ra2.

3. Zaro BW, Yang YY, Hang HC, Pratt MR (2011) Chemical reporters for fluorescent detection and identification of O-GlcNAc-modified proteins reveal glycosylation of the ubiquitin ligase NEDD4-1. Proc Natl Acad Sci U S A 108: 8146-8151.

4. Teo CF, Ingale S, Wolfert MA, Elsayed GA, Not LG, et al. (2010) Glycopeptide-specific monoclonal antibodies suggest new roles for O-GlcNAc. Nat Chem Biol 6: 338-343.

5. Wang Z, Pandey A, Hart GW (2007) Dynamic interplay between O-linked N-acetylglucosaminylation and glycogen synthase kinase-3-dependent phosphorylation. Mol Cell Proteomics 6: 1365-1379.

6. Alfaro JF, Gong CX, Monroe ME, Aldrich JT, Clauss TR, et al. (2012) Tandem mass spectrometry identifies many mouse brain O-GlcNAcylated proteins including EGF domain-specific O-GlcNAc transferase targets. Proc Natl Acad Sci U S A 109: 7280-7285.

7. Boyce M, Carrico IS, Ganguli AS, Yu SH, Hangauer MJ, et al. (2011) Metabolic cross-talk allows labeling of O-linked beta-N-acetylglucosamine-modified proteins via the N-acetylgalactosamine salvage pathway. Proc Natl Acad Sci U S A 108: 3141-3146.

8. Khidekel N, Ficarro SB, Clark PM, Bryan MC, Swaney DL, et al. (2007) Probing the dynamics of O-GlcNAc glycosylation in the brain using quantitative proteomics. Nat Chem Biol 3: 339-348.

9. Khidekel N, Ficarro SB, Peters EC, Hsieh-Wilson LC (2004) Exploring the O-GlcNAc proteome: direct identification of O-GlcNAc-modified proteins from the brain. Proc Natl Acad Sci U S A 101: 13132-13137.

10. Vosseller K, Trinidad JC, Chalkley RJ, Specht CG, Thalhammer A, et al. (2006) O-linked N-acetylglucosamine proteomics of postsynaptic density preparations using lectin weak affinity chromatography and mass spectrometry. Mol Cell Proteomics 5: 923-934.

11. Wang Z, Udeshi ND, O'Malley M, Shabanowitz J, Hunt DF, et al. (2010) Enrichment and site mapping of O-linked N-acetylglucosamine by a combination of chemical/enzymatic tagging, photochemical cleavage, and electron transfer dissociation mass spectrometry. Mol Cell Proteomics 9: 153-160.

12. Yang WH, Kim JE, Nam HW, Ju JW, Kim HS, et al. (2006) Modification of p53 with O-linked N-acetylglucosamine regulates p53 activity and stability. Nature cell biology 8: 1074-1083.

13. Nandi A, Sprung R, Barma DK, Zhao Y, Kim SC, et al. (2006) Global identification of O-GlcNAc-modified proteins. Anal Chem 78: 452-458.

14. Trinidad JC, Barkan DT, Gulledge BF, Thalhammer A, Sali A, et al. (2012) Global identification and characterization of both O-GlcNAcylation and phosphorylation at the murine synapse. Mol Cell Proteomics 11: 215-229.

15. Clark PM, Dweck JF, Mason DE, Hart CR, Buck SB, et al. (2008) Direct in-gel fluorescence detection and cellular imaging of O-GlcNAc-modified proteins. J Am Chem Soc 130: 11576-11577.

16. Dehennaut V, Slomianny MC, Page A, Vercoutter-Edouart AS, Jessus C, et al. (2008) Identification of structural and functional O-linked N-acetylglucosamine-bearing proteins in Xenopus laevis oocyte. Mol Cell Proteomics 7: 2229-2245.

17. Gurcel C, Vercoutter-Edouart AS, Fonbonne C, Mortuaire M, Salvador A, et al. (2008) Identification of new O-GlcNAc modified proteins using a click-chemistry-based tagging. Anal Bioanal Chem 390: 2089-2097.

18. Sprung R, Nandi A, Chen Y, Kim SC, Barma D, et al. (2005) Tagging-via-substrate strategy for probing O-GlcNAc modified proteins. J Proteome Res 4: 950-957.

19. Cieniewski-Bernard C, Bastide B, Lefebvre T, Lemoine J, Mounier Y, et al. (2004) Identification of O-linked N-acetylglucosamine proteins in rat skeletal muscle using two-dimensional gel electrophoresis and mass spectrometry. Mol Cell Proteomics 3: 577-585.

20. Ohn T, Kedersha N, Hickman T, Tisdale S, Anderson P (2008) A functional RNAi screen links O-GlcNAc modification of ribosomal proteins to stress granule and processing body assembly. Nature cell biology 10: 1224-1231.

21. Walgren JL, Vincent TS, Schey KL, Buse MG (2003) High glucose and insulin promote O-GlcNAc modification of proteins, including alpha-tubulin. American journal of physiology Endocrinology and metabolism 284: E424-434.

22. Wells L, Vosseller K, Cole RN, Cronshaw JM, Matunis MJ, et al. (2002) Mapping sites of O-GlcNAc modification using affinity tags for serine and threonine post-translational modifications. Mol Cell Proteomics 1: 791-804.

23. Wang Z, Park K, Comer F, Hsieh-Wilson LC, Saudek CD, et al. (2009) Site-specific GlcNAcylation of human erythrocyte proteins: potential biomarker(s) for diabetes. Diabetes 58: 309-317.
